# Minimizing total weighted latency in home healthcare routing and scheduling with patient prioritization

**DOI:** 10.1007/s00291-023-00713-3

**Published:** 2023-03-25

**Authors:** Vahid Akbari, İhsan Sadati, F. Sibel Salman, Davood Shiri

**Affiliations:** 1 Nottingham University Business School, University of Nottingham, Jubilee Campus, Nottingham, NG8 1BB UK; 2 Faculty of Engineering and Natural Sciences, Sabanci University, Istanbul, Turkey; 3 Smart Mobility and Logistics Lab, Sabanci University, Istanbul, Turkey; 4 College of Engineering, Koç University, 34450 Sariyer, Istanbul Turkey; 5 Sheffield University Management School, University of Sheffield, Conduit Road, Sheffield, S10 1FL UK

**Keywords:** Home healthcare, Personnel routing, Prioritized patient scheduling, Multiple traveling repairman problem, Variable neighborhood search

## Abstract

We study a home healthcare routing and scheduling problem, where multiple healthcare service provider teams should visit a given set of patients at their homes. The problem involves assigning each patient to a team and generating the routes of the teams such that each patient is visited once. When patients are prioritized according to the severity of their condition or their service urgency, the problem minimizes the total weighted waiting time of the patients, where the weights represent the triage levels. In this form, the problem generalizes the multiple traveling repairman problem. To obtain optimal solutions for small to moderate-size instances, we propose a level-based integer programming (IP) model on a transformed input network. To solve larger instances, we develop a metaheuristic algorithm that relies on a customized saving procedure and a general variable neighborhood search algorithm. We evaluate the IP model and the metaheuristic on various small-, medium- and large-sized instances coming from the vehicle routing literature. While the IP model finds the optimal solutions to all the small- and medium-sized instances within three hours of run time, the metaheuristic algorithm achieves the optimal solutions to all instances within merely a few seconds. We also provide a case study involving Covid-19 patients in a district of Istanbul and derive insights for the planners by means of several analyses.

## Introduction

Home healthcare services have been growing globally (Euchi et al. 2022; Cinar et al. 2021), mostly due to the increase in life expectancy, as well as the increase in the number of patients with chronic diseases and physical disabilities (Fikar and Hirsch 2017; Tippong et al. 2022). These services not only improve the quality of service provided to the patients but also decrease hospital congestion (Grenouilleau et al. 2019). The pressure on hospitals could be eased by the delivery of a wide range of services from filiation and testing to drug delivery and wound treatment at home. For elderly and disabled patients, vaccination could also be conducted at their homes. While the range of home healthcare services is wide, our problem definition and case study are based on optimizing the routes and schedules of teams that provide filiation services for patients during the pandemic. In our context, filiation services refer to the standard services concerned with assessing the conditions of patients affected by an infectious disease.

In most cases, patients’ health conditions differ and it is essential to prioritize them accordingly. To achieve this, a triage level is defined for each patient that shows the degree of urgency and identifies the severity of the patient’s condition. These levels are used as weights for the waiting times (latency) of the patients in the objective function of our problem so that urgent patients are more likely to get service sooner. Henceforth, we refer to this routing and scheduling problem as the *Home Healthcare Routing and Scheduling Problem with Patient Prioritization* (HHRSP-PP).

The HHRSP-PP is closely related to the traveling repairman problem (TRP) and its variants that have been studied in the routing literature. In fact, when the service time of a node (e.g., patient) is added to the traveling time of each incoming arc to that node, the HHRSP-PP can be viewed as a generalization of the multiple traveling repairman problem (mTRP) where the waiting time of each node is multiplied by its priority weight. Recently, the “weighted multiple traveling repairman problem” (WmTRP) has been studied by Muritiba et al. (2021). The authors investigated an application of the WmTRP related to the maintenance of speed cameras, where each node should be visited by only one repairman. In our application, the weight of each node (patient) is associated with the triage level, which is an integer number, typically between 1 and 5.

While the HHRSP-PP and the WmTRP are similar in terms of problem definition, our methodological approach to deal with the problem is different from the approach presented in Muritiba et al. (2021). As opposed to a standard mixed integer programming (MIP) formulation that is only capable of providing exact solutions to very small-sized instances, we develop an alternative level-based integer programming (IP) model, together with valid constraints, by means of which medium-sized instances are solved optimally in reasonable run times. We compare the level-based IP model with the standard MIP model on small-sized instances with 10 and 20 patients (nodes) adopted from the TRP literature (Salehipour et al. 2011).

In order to solve larger instances, we develop a metaheuristic algorithm in which the initial solutions are generated using a problem-specific saving method. A generalized variable neighborhood search (GVNS) procedure is then employed at the improvement step. We test our heuristic algorithm and the level-based IP model on several sets of instances adapted from: (i) the TRP literature (Salehipour et al. 2011), (ii) the vehicle routing problem (VRP) literature (Augerat et al. 1995) and (iii) the WmTRP instances provided in Muritiba et al. (2021). In all the tested instances with up to 60 patients and 15 healthcare service provider teams (HSPTs), optimal solutions are found by both solving the level-based IP model and the proposed heuristic algorithm. However, the metaheuristic algorithm runs within merely 9 s. The performance of our algorithm is further tested on larger instances and compared with well-known algorithms applied to instances of the TRP and the mTRP. We note that our instances and obtained solutions can be used for benchmarking in future studies.

A case study based on the COVID-19 filiation services provided at patient homes in the Kağıthane district of Istanbul is also presented. The data consists of 647 prioritized patients who are clustered into nine regions so that an HSPT is responsible for patients in each cluster. The solution shows that patient prioritization enables patients with more severe conditions to be serviced earlier. A comparison with a centralized optimization approach for a multi-depot system where patients are not clustered prior to optimization reveals how much the centralized system improves both the quality of the solutions and the CPU running times.

The rest of the paper is organized as follows. A review of the related literature is presented in Sect. 2, and the mathematical models are provided in Sect. 3. The metaheuristic algorithm is described in Sect. 4. The results of the experimental study that tests the models and the metaheuristic algorithm are given in Sect. 5. The case study is presented in Sect. 6. Finally, concluding remarks and future work directions are stated in Sect. 7.

## Literature review

In this section, we review studies related to home healthcare routing and scheduling, as well as the TRP and its variants.

### Home healthcare routing and scheduling

The number of studies addressing home healthcare routing and scheduling has been growing rapidly in recent years. Comprehensive reviews of studies in the context of home healthcare routing and scheduling are provided in Euchi et al. (2022), Grieco et al. (2021), Fikar and Hirsch (2017) and Cisse et al. (2017). Here, we focus on studies that are similar to ours from several perspectives. From the planning horizon viewpoint, the studies on this topic are classified as single- and multi-period. Multi-period models are suitable for problems where a large number of requests are received a priori and must be served on a long-term planning horizon. In this case, the visits must be scheduled over multiple working shifts (Fikar and Hirsch 2017; Bowers et al. 2015). On the other hand, the single-period models are a better fit for problems where all the requests that are accumulated at the beginning of the working shift must be handled within the upcoming shift. In our study, we consider a single-period planning horizon during which all the patients should be visited in the upcoming working shift. Accordingly, the focus of the literature review in this section revolves around single-period studies.

The constraints defined typically in single-period models are time window requirements of the patients, skill requirements, working time regulations and precedence. However, the majority of studies use soft time windows (e.g., Trautsamwieser et al. 2011; Eveborn et al. 2006; Bertels and Fahle 2006) and require all patients to be visited (e.g., Liu et al. (2013); Cappanera et al. (2018)). Given that time windows and latency objectives are in conflict, we do not consider time windows for the patients. Furthermore, we focus on the case with homogeneous teams and consider healthcare service provider teams that are self-contained and can provide all the required services.

The studied problems in the literature can also be categorized as deterministic or stochastic, where stochastic studies address the uncertainty in service times or travel times. Since patients conditions are known a priori in our problem, we assume the service times can be predicted accurately. Thus, our optimization problem has a deterministic nature. As a result, here we focus on deterministic single-period problems. Few studies in this group provide exact solution methods such as branch-and-price (e.g., Rasmussen et al. 2012; Manerba and Mansini 2016), and most focus on heuristic approaches. Variable neighborhood search (VNS) (e.g., Mankowska et al. 2014; Trautsamwieser et al. 2011), memetic algorithm (e.g., Decerle et al. 2018), simulated annealing (e.g., Hiermann et al. 2015), genetic algorithm (e.g., Li et al. 2021), particle swarm optimization (e.g., Akjiratikarl et al. 2007), set partitioning heuristics (e.g., Grenouilleau et al. 2019) and quantitative threshold-based approaches (e.g., Nasir and Dang 2020) are among the heuristics developed.

Various objective functions have been considered in the home healthcare routing and scheduling literature, including the minimization of the travel time (e.g., Bredstrom and Ronqvist 2008; Trautsamwieser et al. 2011; Hiermann et al. 2015), travel cost (e.g., Eveborn et al. 2006; Bertels and Fahle 2006; Rasmussen et al. 2012), waiting time of service provider teams (e.g., Trautsamwieser et al. 2011), overtime (e.g., Hiermann et al. 2015; Trautsamwieser et al. 2011), maximization of the preferences (e.g., Bertels and Fahle 2006; Bredstrom and Ronqvist 2008; Trautsamwieser et al. 2011; Rasmussen et al. 2012; Hiermann et al. 2015) and balancing the workload among the HSPTs (e.g., Cappanera et al. 2018; Manerba and Mansini 2016). We depart from these studies by minimizing the weighted total latency of the patients. This type of objective is known to be more challenging than the standard objective of minimizing the total tour length in the routing literature (Angel-Bello et al. 2013). However, in healthcare, waiting can be critical for some patients, and forming routes that minimize the total weighted waiting times carries particular importance in this context. To the best of our knowledge, our article is the first paper that considers the latency objective for a home healthcare routing and scheduling problem.

### Traveling repairman problem and its variants

The traveling repairman problem (TRP) aims to keep the customers wait less in a service setting. While the name comes from the repair services, alternative names have also been given by some researchers. In the TRP, a complete graph with a depot node and positive edge distances are provided. The repairman has to perform a route starting from the depot node, visiting all the nodes and ending at the depot node with the objective of minimizing the summation of all visit times. In our problem, the HSPTs must visit all the patients. Moreover, the service time of each patient is incorporated by adding the service time to the traveling time of the incoming arcs to that patient. Since we consider a weighted latency objective function, our problem can be considered as a generalization of the TRP where weights are associated with the customer nodes and multiple repairmen are considered, as opposed to the TRP, which optimizes the route of a single repairman. We next present a brief overview of the previous work on the TRP and its variations.

Numerous articles proposed exact solution procedures including mathematical models for the TRP (e.g., see Sarubbi et al. 2008; Picard and Queyranne 1978; Mendez-Diaz et al. 2008). Mendez-Diaz et al. (2008) formulated a three-index mathematical model that can solve instances having up to 40 nodes by taking advantage of effective valid constraints. Bulhões et al. (2018) developed a branch-and-price algorithm for a set partitioning formulation. Angel-Bello et al. (2013) designed two mathematical models for the TRP based on a multi-level network system. Based on their computational study, Angel-Bello et al. (2013) conclude that level-based models outperform other modeling approaches for the TRP. On the other hand, several studies have developed heuristics and metaheuristics to solve the TRP. Salehipour et al. (2011) developed two metaheuristics where the initial solutions are generated using a greedy randomized adaptive search procedure (GRASP). They have used VNS and variable neighborhood descent (VND) for the improvement phase of their metaheuristics. They called their algorithms the GRASP+VNS and GRASP+VND algorithms. Mladenović et al. (2013) developed a VNS algorithm for the TRP. They tested their algorithm on instances provided in Salehipour et al. (2011) and showed that their proposed algorithm outperforms the GRASP+VNS and GRASP+VND algorithms on these instances.

The multiple TRP (mTRP) is a generalization of the TRP in the sense that it involves finding the routes of multiple repairmen. Luo et al. (2014), Nucamendi et al. (2015), and Nucamendi-Guillén et al. (2016) provided different methods to address the mTRP. For example, Nucamendi-Guillén et al. (2016) proposed a metaheuristic algorithm that contains an iterated greedy (IG) phase for initiation and a local search (LS) phase for improvement. Sze et al. (2017) developed an adaptive variable neighborhood search algorithm to solve the mTRP. Angel-Bello et al. (2019), on the other hand, focused on exact approaches and proposed five mathematical models for the mTRP. The first three formulations emanated from the classical flow-based formulations and the latter two were based on time-dependent models regarding a multi-level network. These models perform much better than the first three. Liu et al. (2018) developed a branch-and-price algorithm to solve the multi-trip variation of the mTRP with up to 85 nodes. More recently, larger instances were solved exactly and approximately; Bruni et al. (2022) studied the multi-depot mTRP and proposed two mathematical models as well as a hybrid genetic algorithm to solve instances with up to 240 nodes.

Several authors proposed heuristic algorithms for various variations of the mTRP. Bang (2018) considered a variation of the mTRP with a distance constraint, where each vehicle is not permitted to travel longer than a given limit. They designed a GRASP heuristic to generate the initial solution, and a VND algorithm to improve the initial solution. In Avci and Avci (2019), the authors designed an adaptive large neighborhood search algorithm (ALNS) for solving the mTRP with time-dependent profits. Lalla-Ruiz and Voß (2020) addressed the multi-depot extension of the mTRP and proposed a matheuristic algorithm.

While both the TRP and mTRP are well-studied problems, there are only a few studies that focus on the variations of these problems where weights play a role in the objective function. Dewilde et al. (2013) employed a tabu search algorithm for the prize-collecting TRP, where prizes are associated with the nodes and a subset of the nodes should be selected to be visited to collect the maximum total prize within a given time limit. In this variation, a prize of $p_i - t_i$ is collected when the repairman arrives at node $i \in V$ at time $t_i$, where $p_i$ is a fixed prize allocated to node $i\in V$. This variation differs from minimizing the weighted waiting times (latencies) as the problem is selective and the prizes are time-dependent. Lu et al. (2019) developed a memetic algorithm to solve a generalization of the problem introduced in Dewilde et al. (2013) with multiple repairmen. García et al. (2002) provided a linear time exact algorithm and Wu (2000) presented a dynamic programming model for solving a special case of the weighted TRP (WTRP) in which the underlying graph is a path. Akbari and Shiri (2021) addressed an online variation of the weighted TRP considering only one repairman. In a recent study, Muritiba et al. (2021) proposed a branch-and-cut algorithm and developed an iterated local search algorithm to address the weighted multi-repairmen WTRP, motivated by an application involving speed cameras to be maintained.

To the best of our knowledge, none of the TRP variations we have encountered in the literature have been studied in the context of home healthcare. Our paper is the first study that investigates the “latency” objective in this context. That is, we study a TRP with multiple repairmen and a weighted latency objective function, motivated by the home healthcare routing and scheduling problem. Our level-based IP model and metaheuristic algorithm are also new additions to the WmTRP literature.

## Mathematical models

We define the HHRSP-PP on a complete graph $G = (V_0, E)$ with node set $V_0=\{0, 1,\ldots , n\}$ and arc set $A=\{(i,j):i,j \in V_0, i \ne j\}$. The depot (starting location of the HSPTs) is located at node 0 and the set of nodes to be serviced (patients) is represented by $V = V_0 {\setminus } \{0\}$. The distance from node *i* to node *j* (or travel time) is given as $c_{ij}, (i,j) \in A$, according to the locations of patients *i* and *j* and the shortest path in the road network between them. We incorporate service times in our problem by adding the service time of patient *i* ($i \in \{1,2,\ldots ,n\})$ to the traveling time of each incoming arc to patient *i*. Since each patient must be visited exactly once, the optimal solution and the optimal objective function value remain unchanged after this transformation. A priority (triage level or weight) is associated with each patient (i.e., each node in *V*) that is denoted by $w_i, i \in V$, where higher values indicate increased priority. In the HHRSP-PP, there are *m* number of HSPTs that originate from the depot node at time $0, (t_0 = 0)$, traverse a number of nodes to service the patients, and return back to the depot. The collection of the routes of the HSPTs should be such that each patient in *V* is serviced in one of the routes. Clearly, it can be assumed that the number of HSPTs is less than the number of patients that should be serviced, i.e., $m < n$. Letting $t_i$ be the time at which patient $i\in V$ is visited, the objective function of the HHRSP-PP is to minimize $\sum _{i\in V} w_i t_i$. Next, we present two formulations for this problem.

### Model I

Model I is based on calculating the arrival time to each patient considering the visit time of the previously serviced patients by the same HSPT and the travel time between the patients and also the service time of the considered patient. Since the visit time of a patient must be greater than the time at which the previous patient was visited, the solution cannot contain a closed tour. However, the optimal solution of the problem should include *m* closed tours starting and ending in the depot node, where *m* shows the number of HSPTs. In order to remedy this issue, a dummy sink node indexed by $n+1$ is defined such that the HSPTs finish their routes at this node. For this purpose, we set the time of going from $i \in V$ to the dummy sink node equal to the time of going from *i* to the depot excluding the service time of node *i*. We show the union of the node set *V* together with the sink node $n+1$ by $V_{n+1} = V \cup \{n+1\}$ and the union of all nodes consists of the depot and the sink node by $V_{0,n+1}$. In this model, $x_{ij} \in \{0,1\} \; (i \in V_0, j \in V_{n+1}, i \ne j)$ is the first set of variables that denote whether a HSPT is going from node *i* to node *j* or not, and $t_i \ge 0 \; (i \in V_{0,n+1})$ is the second set of variables that denote the time at which patient *i* is visited.

1 $$\begin{aligned}&{\textbf {Min}} \quad \sum _{i\in V} w_i t_i \end{aligned}$$

2 $$\begin{aligned}&\sum _{i\in V_{n+1}} x_{0i} = m \end{aligned}$$

3 $$\begin{aligned}&\sum _{i\in V} x_{in+1} = m \end{aligned}$$

4 $$\begin{aligned}&\sum _{j\in V_{n+1} {\setminus } \{i\}} x_{ij} = 1, \quad i \in V \end{aligned}$$

5 $$\begin{aligned}&\sum _{i \in V_0 {\setminus } \{j\}} x_{ij} = 1, \quad j \in V \end{aligned}$$

6 $$\begin{aligned}&t_j \ge t_i + c_{ij} x_{ij} - M(1-x_{ij}), \quad i \in V_0, j\in V_{n+1}, i\ne j \end{aligned}$$

7 $$\begin{aligned}&x_{ij} \in \{0,1\}, \quad i \in V_0, j \in V_{n+1}, i \ne j\end{aligned}$$

8 $$\begin{aligned}&t_i \ge 0, \quad i \in V_{0,n+1} \end{aligned}$$

The objective function in (1) minimizes the total weighted latency (arrival times) of all the patients nodes. Constraints (2) and (3) ensure that *m* routes are determined for the *m* HSPTs. Constraints (4) and (5) ensure that each patient is visited exactly once. Constraints (6) calculate the arrival times and prevent sub-tours. In this constraint, *M* can get any value greater than or equal to $\sum _{(i,j) \in A} c_{ij}$.

### Model II

As mentioned, since each patient must be visited exactly once, the HHRSP-PP is equivalent to the WmTRP when the service times of patients are added to the traveling times of incoming arcs to the corresponding patient nodes. Model II is based on a multi-level model developed in Angel-Bello et al. (2019) for the multiple TRP with no weights. We modify the mathematical formulation of Angel-Bello et al. (2019) to make it applicable to HHRSP-PP.

**Fig. 1:**
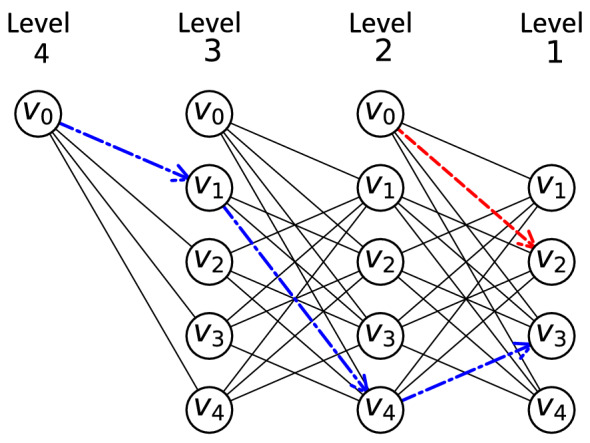
Level-based structure

Angel-Bello et al. (2019) showed computationally that a level-based model outperforms flow-based (Gavish and Graves 1978) or multiple TSP-based (Bektas 2006) formulations for latency objectives in terms of computational time. This is because the level-based model is designed to compute the arrival times to the patients (nodes) via the objective function. In Model II, a set of binary variables $x_{ij}^r$ are defined where if the arc (*i*, *j*) is used to link patient *i* at level $r+1$ with patient *j* at level *r*, then it will be equal to one. Levels in this model are defined to facilitate the modeling and show the steps of the movements for each of the HSTPs. An illustration of the level-based model is provided in Fig. 1. In this example, a case with four patients ($n=4$) and 2 HSPTs ($m=2$) is considered. Since each HSPT visits at least one patient in the optimal solution, the number of levels at which a patient is visited is restrained by $L = n - m + 1$, which is equal to 4 in our example. A sample route for each of the HSPTs is also presented in Fig. 1. The route of one of the HSPTs include visiting patients 1, 4 and 3 consecutively. For this HSPT, the teams leave the depot in level 4, visits patient $v_1$ in level 3 and then visits patients $v_4$ and $v_3$ in levels 2 and 1, respectively. For the other HSPT, they only meet patient $v_2$ in level 1.

**Fig. 2:**
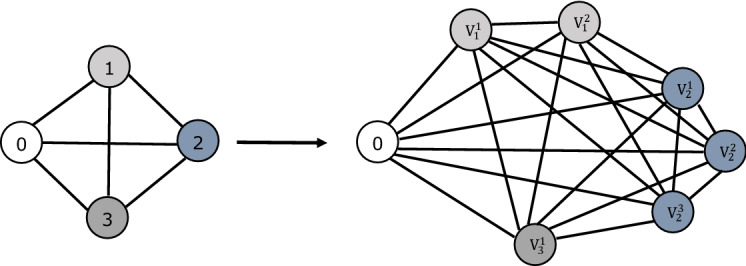
Demonstration of the transformation

Given that this level-based formulation was first proposed for the case with no weights on the nodes (patients), to develop Model II we define a transformation of the input graph $G = (V_0, E)$ to a graph denoted by $\mathcal {G} = (\mathcal {V}_0, \mathcal {E})$. Suppose that the weight (triage level) $w_i$ for a node (patient) *i* in *V* is an integer number which represents the severity of the condition of patient *i*. $\mathcal {G}$ is a complete graph with node set $\mathcal {V}_0$ that includes the depot and $w_i$ *duplicated* nodes for each node $i \in V$. An example of this transformation is given in Fig. 2. In this example, there are three patients that should be visited. The triage level of the first patient is two $(w_1 = 2)$, the triage level of the second patient is three ($w_2 = 3$), and the triage level of the third patient is one $(w_3 = 1)$. In this transformation of the graph *G* to graph $\mathcal {G}$, each node $i\in V$ with a weight of $w_i$ is replaced with $w_i$ duplicated nodes denoted by $V_i^a: i \in V, \ a \in \{1,\ldots ,w_i\}$. As a result, $\mathcal {G}$ is a complete graph with node set $\mathcal {V}_0 = 0 \cup \{V_i^a: i\in V, \ a \in \{1,\ldots ,w_i\}\}$. Furthermore, the set of nodes in $\mathcal {G}$ excluding the depot is denoted by $\mathcal {V}$.

In graph $\mathcal {G}$, the cost or distance of going from node $V_i^a$ to node $V_j^b$ is set equal to $c_{ij}$ for $i, j \in V_0$ if $i \ne j$, $a \in \{1,\ldots ,w_i\}$ and $b \in \{1,\ldots , w_j\}$. If $i = j$ for two nodes in $\mathcal {V}$ (i.e., if both nodes are duplicated nodes of the same patient), then the cost of moving between them equals 0.

#### Proposition 1

*The optimal solution and its objective function value on* $\mathcal {G}$ *is the same as the optimal solution and its objective function value on* *G*.

#### Proof

First, let us assume the optimal visiting times on *G* are given as $t_i^*$ for $i \in V$. As a result, $\sum _{i\in V} w_it_i^*$ represents the optimal objective function value on *G*. The distance from $V_i^a \in \mathcal {V}$ to $V_j^b \in \mathcal {V}$ on $\mathcal {G}$ equals the distance from $i \in V$ to $j \in V$ on *G* for $i \ne j$. Also, the distance from $V_i^a \in \mathcal {V}$ to $V_j^b \in \mathcal {V}$ equals 0 when $i=j$. Thus, we transform the optimal solution obtained on *G* to a feasible solution on $\mathcal {G}$ such that $t_{V_i^a} = t_i^*$ for $i \in V$ and $a\in \{1,\ldots ,w_i\}$. Noting this, we can rewrite $\sum _{i\in V} w_it_i^*$ as $\sum _{a=1}^{w_i} \sum _{i\in V} t_{V_i^a}$. This corresponds to finding a feasible solution on $\mathcal {G}$ with the same objective function value obtained on *G*.

There exists an optimal solution on $\mathcal {G}$ such that once one of the $w_i$ duplicated nodes of patient *i* is visited by one HSPT, all the other ${w_i} -1$ duplicated nodes of patient *i* will be visited before that HSPT leaves patient *i*. This is because the distances between the duplicated nodes for the same patient are set equal to zero and once a HSPT arrives to a duplicated node of a patient, the visiting time of all the other duplicated nodes of that patient is set to that time. This means that an optimal solution on $\mathcal {G}$ corresponds to a feasible solution on *G* with the same objective function value and the proof is completed. $\square$

Relying on Proposition 1, we develop a level-based model to solve the HHRSP-PP on $\mathcal {G}$ as described in the following. In this model, we only have $x_{V_i^aV_j^b}^r$ variables that are equal to 1 if arc $(V_i^a,V_j^b)$ is used by a HSPT to visit node $V_j^b$ at level *r* immediately after visiting node $V_i^a$ at level $r+1$. In this model, the maximum number of levels is $\mathcal {L} = \sum _{i\in V}{w_i} - m + 1$. For simplicity of notation, indices *u* and *s* are used to show the nodes in set $\mathcal {V}$ as well.

9 $$\begin{aligned}&{\textbf {Min}} \quad \sum _{u\in \mathcal {V}} c_{0u} \sum _{r=1}^{\mathcal {L}} rx_{0u}^{r} + \sum _{u \in \mathcal {V}} \sum _{s \in \mathcal {V} {\setminus } u} c_{us} \sum _{r=1}^{\mathcal {L}-1} rx_{us}^{r} \end{aligned}$$

10 $$\begin{aligned}&\sum _{u\in \mathcal {V}} x_{0u}^1 + \sum _{s\in \mathcal {V}} \sum _{u\in \mathcal {V} {\setminus } s} x_{su}^1 = m \end{aligned}$$

11 $$\begin{aligned}&\sum _{r=1}^{\mathcal {L}} \sum _{u\in \mathcal {V}} x_{0u}^{r} = m \end{aligned}$$

12 $$\begin{aligned}&x_{0u}^{r+1} + \sum _{s \in \mathcal {V} {\setminus } u} x_{su}^{r+1} = \sum _{s \in \mathcal {V} {\setminus } u} x_{us}^r, \quad u \in \mathcal {V} , r\in \{1,\ldots ,\mathcal {L}-2\} \end{aligned}$$

13 $$\begin{aligned}&x_{0u}^{\mathcal {L}} = \sum _{s \in \mathcal {V} {\setminus } u} x_{us}^{\mathcal {L}-1}, \quad u \in \mathcal {V} \end{aligned}$$

14 $$\begin{aligned}&\sum _{r=1}^\mathcal {L} x_{0u}^{r} + \sum _{r=1}^{\mathcal {L} -1 } \sum _{s\in \mathcal {V} {\setminus } u} x_{su} ^ {r} = 1, \quad u\in \mathcal {V} \end{aligned}$$

15 $$\begin{aligned}&x_{us}^r \in \{0,1\}, \quad u \in \mathcal {V}_0, s \in \mathcal {V}, u \ne s, r \in \{1,\ldots ,\mathcal {L}\} \end{aligned}$$

The objective function of the level-based model on $\mathcal {G}$ is expressed in (9). Constraint (10) guarantees that exactly *m* HSPTs terminate their routes at level 1. By constraint (11), the number of HSPTs that leave the depot node is set to *m*. The flow conservation constraints are given by constraints (12) and (13) to ensure the continuity of the paths. Constraint set (14) guarantees that each patient is visited by one and only one HSPT. In the level-based model, this is achieved by ensuring that each patient is active only in one of the levels.

#### Valid constraints

Since there exist $w_i$ duplicated nodes for patient *i* ($i \in \{1,2,\ldots ,n\}$) in the level-based formulation, the size of the input graph grows significantly. Moreover, there may be several optimal solutions where the order of the visits of the patients is optimal, but within the duplicated nodes for each patient, the order of visiting the duplicated nodes changes. To remedy this, we propose a number of valid constraints and add them to our level-based model to cut off the feasible space. This way, we eliminate the alternative optimal solutions that correspond to the same order of visiting the patients but allow different combination of visits between the duplicated nodes for the same patient. For that, we set a rule such that for a fixed patient $i \in V$, the HSPT should first visit the duplicated node $V_i^1$, then $V_i^2$, then $V_i^3$,..., and then $V_i^{w_i}$. It should be noted that this rule does not eliminate the optimal solution from the feasible space and only eliminates repetitive solutions that correspond to the same order of visiting the patients. The valid constraints are given in the following.

16 $$\begin{aligned}&\sum _{r=1}^{\mathcal {L}} \sum _{i\in V} x_{0V_i^1}^r = m\end{aligned}$$

17 $$\begin{aligned}&x_{V_i^aV_j^b}^r = 0, \quad i \in V, j \in V, i \ne j, a \in \{1,\ldots ,w_i-1\}, b \in \{2,\ldots ,w_j\}, r\in \{1, \ldots , \mathcal {L} \} \end{aligned}$$

18 $$\begin{aligned}&\sum _{r=1}^{\mathcal {L}} x_{V_i^aV_i^{a+1}}^r = 1, \quad i \in V, a \in \{1,\ldots ,w_i-1\}\end{aligned}$$

19 $$\begin{aligned}&\sum _{j \in V} \sum _{i\in V {\setminus } j} \sum _{r=1} ^ \mathcal {L} x_{V_i^{w_i}V_j^1}^r = n - m \end{aligned}$$

By (16), the *m* HSPTs should visit the first indexed duplicated nodes of *n* patients in *V* as the first node of their route. Constraints (17) ensure that only the first and last indexed duplicated can be visited first and last of a visit to a patient. Given constraints (18), the HSPTs only visit the dummy nodes allocated to a patient in their indexed order. By constraint (19), there are $n-m$ traversals from the last indexed duplicated node of a patient to the first indexed duplicated node of another patient. By adding constraints (16) to (19) to constraints from (9) to (15), we form the second model named as Model II.

## Saving+GVNS heuristic

Model II presented in Sect. 3.2 is shown to solve some moderate-sized instances in our computational tests. This model has two major limitations: firstly, the priority weights (triage levels) of the patients should be integer values and secondly, when the priority weights increase the number of duplicated nodes increases significantly and the problem becomes very difficult to solve. However, here we point out that for our application, the weights of each patient (node) are determined according to triage levels, which are usually integer values between 1 and 5. Nevertheless, in order to overcome these shortcomings, we develop a two-phase algorithm which is capable of handling non-integer weights and is able to solve much larger instances in short time. Similar to Models I and II, we consider the service times in our algorithm by adding the service time of patient *i* ($i \in \{1,2,\ldots ,n\})$ to the traveling time of each incoming arc to the node of patient *i* in the solutions. At the first phase, a saving procedure is used to generate an initial solution. In the second phase, a general variable neighborhood search (GVNS) algorithm is utilized to improve the initial solution found in the first phase. VNS, which has the advantage that it needs few parameters, has been successfully used in solving diverse problems (see Hansen et al. 2008; Pan et al. 2021; Sadati et al. 2022).

Next, an overview of the proposed algorithm, called the Saving+GVNS algorithm, is presented. The algorithm starts with an initial solution $S_0$. A set of neighborhood structures $N_k \quad (k=1,\ldots ,k_{{\textit{max}}})$ are used in the shaking phase and another set of neighborhood structures $M_l \;(l=1,\ldots ,l_{{\textit{max}}})$ in the local search phase. In the shaking phase, a solution $\bar{S}$ is generated by applying the first neighborhood $N_1$ on the initial solution $S_0$. For a given solution $\bar{S}$, local search is performed by applying the first neighborhood $l_1$ to obtain a new solution $S'$. If the new solution $S'$ has a better objective function value compared to the incumbent solution $S^*$, the incumbent solution $S^*$ is updated and local search is continued with the first neighborhood $l_1$. Otherwise, *l* is incremented by 1 and local search is performed using the next neighborhood. This process is continued until all local search neighborhood structures are explored ($l=l_{{\textit{max}}}$). In the local search phase, if a new incumbent solution is produced, index *k* is set to 1. Otherwise, *k* is incremented by 1 and GVNS restarts from the incumbent solution $S^*$. These steps are repeated until a termination criterion is met. The proposed GVNS is terminated when any of the provided conditions is encountered: (i) a specified number of iterations (10,000 in our experiments), (ii) a number of consecutive non-improving iterations (1000 in our experiments). The pseudo-code of the Saving+GVNS algorithm is given as Algorithm 1. Based on line 9 of Algorithm 1, a solution is feasible if it satisfies constraints (2) to (5) of Model I.
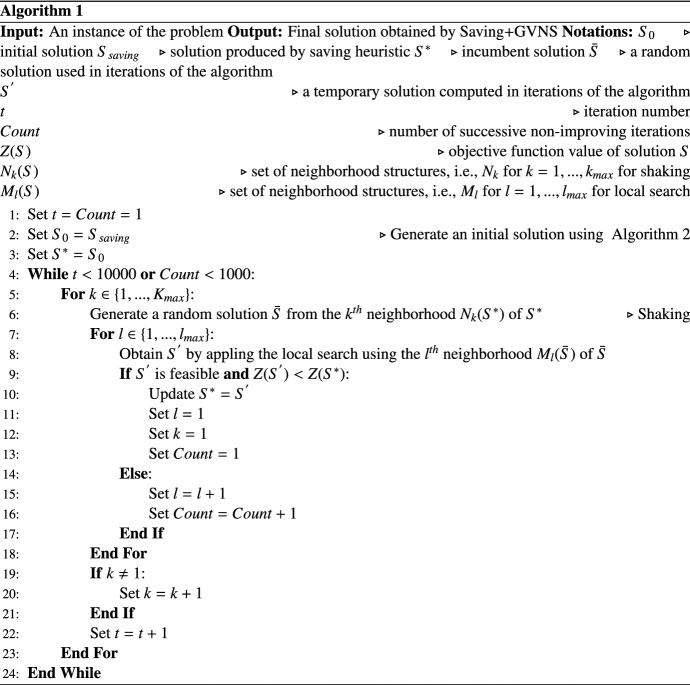


### Generating an initial solution using a saving method

In order to construct a feasible initial solution, we apply a modified greedy savings heuristic which is inspired from the Clarke and Wright (CW) savings algorithm (Clarke and Wright 1964). CW is originally proposed for solving the VRP and contains three steps including; (i) initial allocation of one HSPT to each patient, (ii) calculating the saving from merging two routes and sorting the savings in decreasing order and (iii) merging two routes to form a new route starting from the largest saving if it results in a feasible route. Our proposed saving heuristic is different from the original CW in calculations of the saving values and the feasibility of merging two routes. The saving values in our algorithm are calculated as $S_{ij}=w_j (c_{0j}-c_{0i}-c_{ij})$ where 0 indicates the depot node.

The feasibility of the HHRSP-PP is defined based on using exactly *m* HSPTs. To achieve this, a pre-determined number of patients are assigned to each HSPT. In this regard, at the merging step of the algorithm, at most $\lceil \dfrac{n}{m}\rceil$ patients can be assigned to each HSPT (route) initially, where *n* is the number of patients and *m* is the total number of HSPTs. The pseudo-code of the proposed saving procedure for generating initial solutions is presented in Algorithm 2.
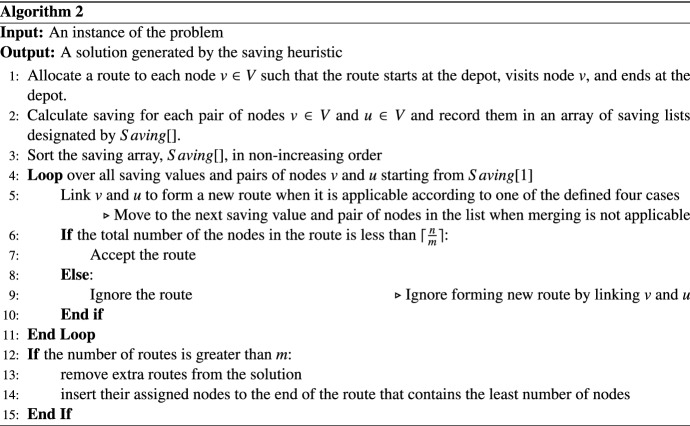


In the merging step at line 5 of Algorithm 2, based on the positions of two given patients *i* and *j*, four cases can be defined as the following:

#### Case I

**Fig. 3:**
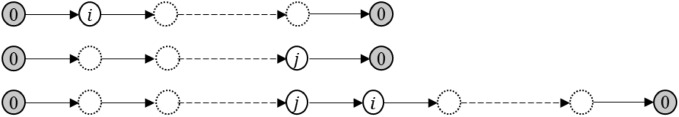
Case I of the merging step

**Fig. 4:**
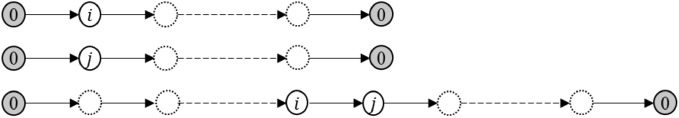
Case II of the merging step

**Fig. 5:**
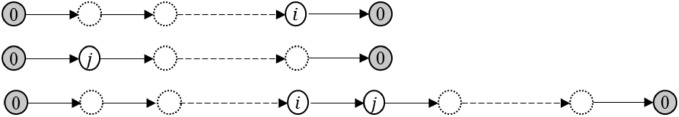
Case III of the merging step

**Fig. 6:**
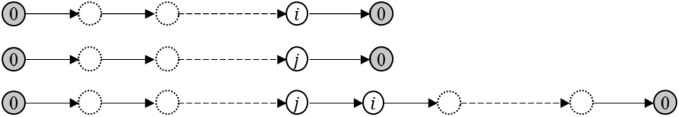
Case IV of the merging step

If patient *i* is the first and *j* is the last patient on two separate routes, edges (0, *i*) and (*j*, 0) will be removed and a new edge (*j*, *i*) will be added to form a single route. Figure 3 gives a visual representation of this merging. Note that in all visual representations of each case (i.e., Figs. 3, 4, 5 and 6), first and second routes give the initial two routes, and the third route shows the merged one.

#### Case II

If patients *i* and *j* are both the first patients on their respective routes, initially the order of the first route is reversed and then edges (*i*, 0) and (0, *j*) are removed and a new route is formed by adding edge (*i*, *j*). Figure 4 gives a visual representation of this merging.

#### Case III

If patient *i* is the last and *j* is the first patient on their respective routes, edges (*i*, 0) and (0, *j*) are eliminated and the merged route is formed by adding (*i*, *j*). Figure 5 gives a visual representation of Case III merging.

#### Case IV

If patients *i* and *j* are both the last patients on their respective routes, initially the order of the first route is reversed and then edges (0, *i*) and (*j*, 0) are removed. Finally, by adding edge (*j*, *i*), the merged route is formed. Figure 6 gives a visual representation of this merging.

If the generated number of HSPTs (routes) *R* at the end of line 11 of Algorithm 2 is greater than *m* (i.e., $m < R$), the extra *R*–*m* routes that include the minimum number of patients are discarded from the solution, and the patients in these routes are inserted to the end of the first *m* routes, which contain the least number of patients (lines 12–14). Note that in line 9 of Algorithm 2, if the new route generated by linking nodes *u* and *v* does not satisfy the assignment of at most $\lceil \dfrac{n}{m}\rceil$ patients, the route is ignored, and the algorithm continues to check other pairs of nodes in the saving list to form a new route (line 4).

### Shaking

**Fig. 7:**
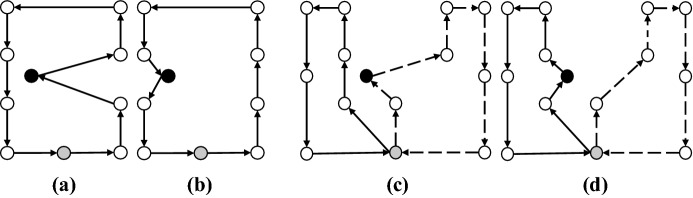
1–0 move: *Same Route:* [**a** Before, **b** After] - *different Routes:* [**c** Before, **d** After]

**Fig. 8:**
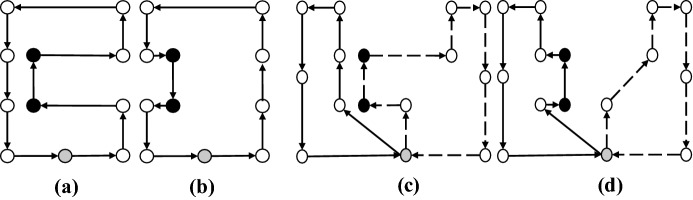
2–0 move: *Same Route:* [**a** Before, **b** After] - *different Routes:* [**c** Before, **d** After]

**Fig. 9:**
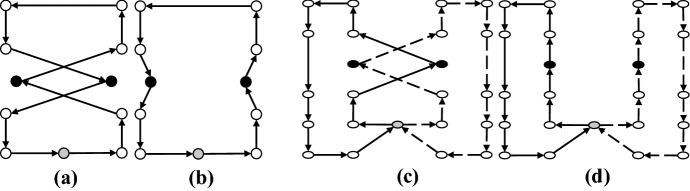
1–1 Exchange: *Same Route:* [**a** Before, **b** After] - *different Routes:* [**c** Before, **d** After]

**Fig. 10:**
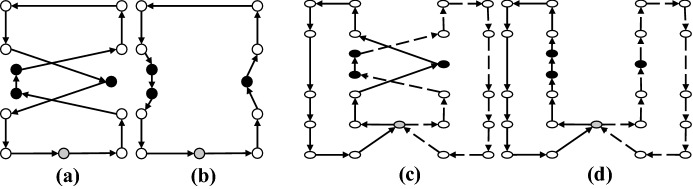
1–2 Move: *Same Route:* [**a** Before, **b** After] - *different Routes:* [**c** Before, **d** After]

**Fig. 11:**
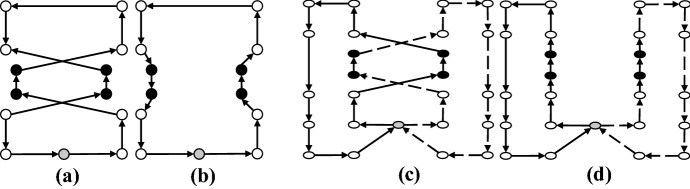
2–2 Exchange: *Same Route:* [**a** Before, **b** After] - *different Routes:* [**c** Before, **d** After]

In the shaking phase, a solution is generated by applying the set of neighborhoods $N_k$. In our implementation, we used five neighborhoods for the shaking phase of our GVNS. These moves are applied both as intra-route and inter-route moves. In Figs. 7, 8, 9, 10 and 11, parts (a) and (b) show the pre-move and post-move states of the intra-route move, respectively, and parts (c) and (d) show the pre-move and post-move states of the inter-routes move for two HSPTs, respectively. In the case where the routes of two HSPTs are considered (parts (c) and (d) of the figures), the routes are distinguished by solid and dashed lines.

*1–0 Move* In this move, a patient from his/her current position is removed and inserted in another new position (Fig. 7).

*2–0 Move* In this move, a patient and his successor are removed and inserted in other new positions (Fig. 8).

*1–1 Exchange* This move swaps the positions of two given patients (Fig. 9).

*1–2 Move* For two given patients, this move swaps the position of the first patient with the second patient and her successor (Fig. 10).

*2–2 Exchange* For two given patients, this move swaps the positions of the first patient and his successor with the second patient and her successor (Fig. 11).

The aforementioned neighborhoods are implemented both as intra- and inter-route moves and are explored in a cyclic sequential order starting with $N_1 = 1$–0 move and ending with $N_5 = 2$–2 Exchange.

### Local search

At each iteration of the GVNS, local search is performed by applying the set of four move operators. In these moves, instead of changing the position of the patients, the steps of the traversed edges are modified.

**Fig. 12:**
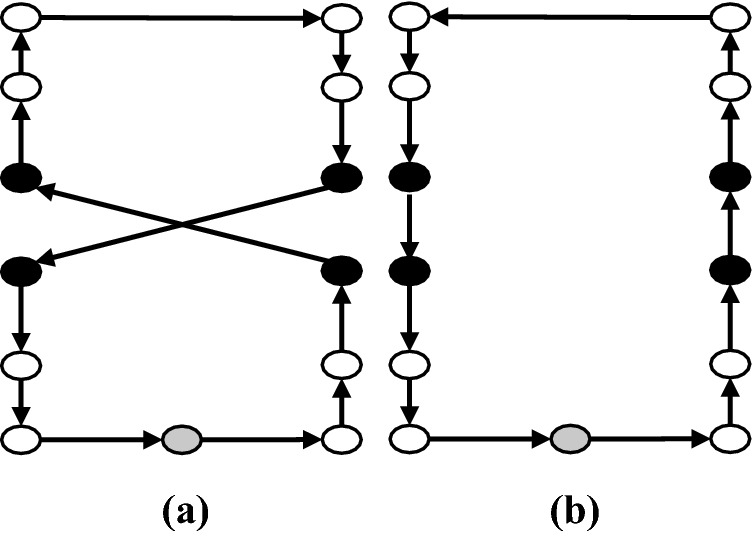
**a** Before 2-Opt move, **b** after 2-Opt move

*2-Opt* For a given set of two arcs in a single route that define a crisscross, this move substitutes them with two new arcs by reversing the sequence of the nodes visited in between (Fig. 12).

**Fig. 13:**
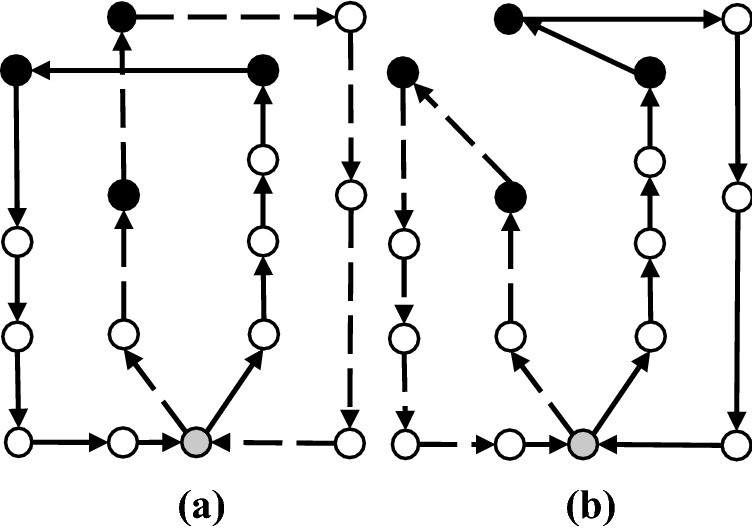
**a** Before 2-Opt* move, **b** after 2-Opt* move

2-Opt* This move is a modification of the 2-Opt move. For a given set of two arcs from two separate routes that create a crisscross, they are replaced with two new edges without reversing the sequence of the patients (Fig. 13).

**Fig. 14:**
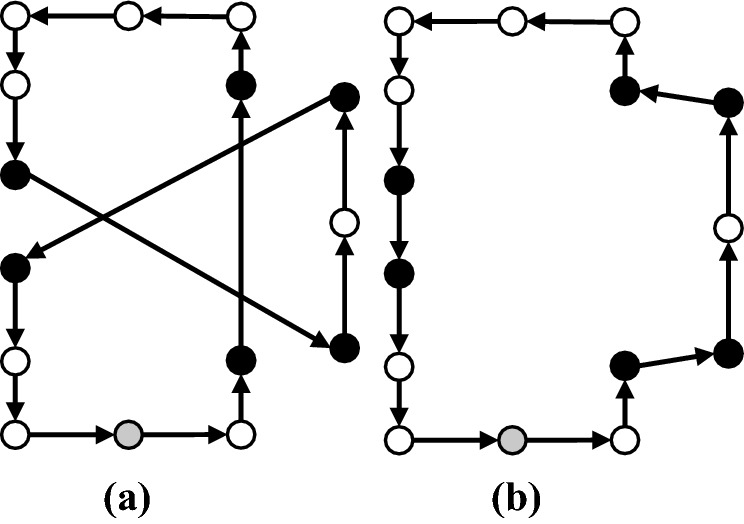
**a** Before 3-Opt move, **b** after 3-Opt move

*3-Opt* In this move, three arcs in a given route are removed and then the network is reconnected in all feasible ways (Fig. 14).

**Fig. 15:**
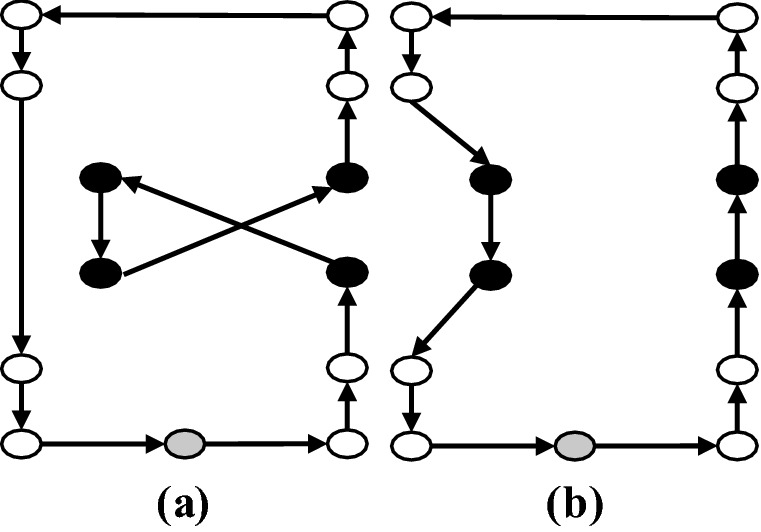
**a** Before Or-Opt move, **b** after Or-Opt move

*Or-Opt* This move resettles a chain of successive patients by substituting three arcs with three new ones without reversing the sub-routes (Fig. 15).

Here, we point out that 2-Opt, 3-Opt and Or-Opt are applied only as intra-route moves. In these four move operators of local search, we resort to the best improvement approach. These neighborhoods are investigated in a cyclic sequential ordering beginning from $M_1= 2$-Opt and terminating with $M_4= \textrm{Or}$-Opt. If a new and better solution is produced by applying any of these neighborhood structures, the local search is continued with the first neighborhood $M_1= 2$-Opt (i.e., the index *l* is reinitialized to one). Otherwise, the local search is performed using the next neighborhood. This process is continued until all local search neighborhood structures are explored (lines 7–18 of Algorithm 1).

## Computational study

For the purpose of conducting a comprehensive computational experiment, we compare the performance of the proposed Saving+GVNS algorithm with the best known algorithms from the literature for various variants of the WmTRP. All experiments were performed on a computer equipped with Intel Xeon(R) and 3.60 GHz processor. The models were coded in Python and solved using Gurobi Optimizer 9 with an academic license. The Saving+GVNS algorithm was coded in C# Visual Studio 2019.

### Performance of the Saving+GVNS algorithm

In this section, we assess the performance of the Saving+GVNS algorithm on simplified versions of WmTRP from the literature. In Sects. 5.1.1 and 5.1.2, our algorithm is compared with TRP and mTRP instances from the literature, respectively.

#### Performance of the Saving+GVNS on TRP instances

In this section, we analyze the performance of Saving+GVNS algorithm on a TRP variation. This version of the TRP has three main differences with WmTRP: (i) nodes are not weighted and hence the weight of each node is set to one, (ii) only one repairman exists, and (iii) the time in which the repairman returns to the depot is added to the objective function. Moreover, the authors of this study compared their results with the Branch-and-Cut-and-Price (B&C&P) algorithm tested on the TSPLib instances presented in Abeledo et al. (2013). We selected those TSPLib instances that were tested in Mladenović et al. (2013) and utilized two-dimensional Euclidean distances. The results of the experiments are given in Table 1.

In Table 1, the name of the instance is given in the “Instance” column. The objective function values obtained in Abeledo et al. (2013) and Mladenović et al. (2013) were the same in these instances and they are given in the second column. These values are directly extracted from these articles. Those values that are given with a bold font are the optimal solutions of the corresponding instances. The results of the Saving+GVNS algorithm on these instances are given in the next columns. In the presented tables, OFV denotes objective function value and CRT refers to the CPU run time in seconds. Since the VNS of Mladenović et al. (2013) was able to find similar solutions to the B&C&P in all the instances, we only reported the gap between the best found objective function value from the Saving+GVNS algorithm and the results reported in the literature. Since these algorithms were not coded by us and were tested on computers prior to 2013, we have not reported their CPU run time. In total, in nine instances out of 12, optimal solutions were found using the Saving+GVNS algorithm. The maximum gap was in instance rat99 with 1.01% and the average gap over these instances was 0.18%. The average CPU run time over these instances was 132.70 s with a maximum of 246.34 s for the lin105 instance. Considering that the Saving+GVNS algorithm was developed to solve the WmTRP and is now tested on a special case of the WmTRP, obtaining such small gaps is promising and verifies the good performance and robustness of the Saving+GVNS algorithm in terms of both the solution quality and CPU run time.

**Table 1:** Results of Saving+GVNS on TSPlib instances

Instance	Abeledo et al. (2013)	Saving+GVNS		
	Mladenović et al. (2013)	Best OFV	CRT	GAP (%)
eil51	**10,178**	**10,178**	15.87	0.00
berlin52	**143,721**	**143,721**	18.25	0.00
st70	**20,557**	**20,557**	60.87	0.00
eil76	**17,976**	**17,976**	81.15	0.00
pr76	**3,455,242**	3,464,384	82.31	0.26
rat99	57,986	58,571	135.14	1.01
kroA100	**983,128**	**983,128**	146.31	0.00
kroB100	**986,008**	**986,008**	150.49	0.00
kroC100	**961,324**	966,106	147.79	0.50
kroD100	976,965	981,728	191.55	0.49
kroE100	**971,266**	**971,266**	165.22	0.00
rd100	**340,047**	340,706	203.01	0.19
eil101	27,513	27,570	173.11	0.21
lin105	**603,910**	**603,910**	246.34	0.00
pr107	**2,026,626**	**2,026,626**	173.05	0.00
Average			132.70	0.18

#### Performance of the Saving+GVNS on mTRP instances

In this section, we test the performance of the Saving+GVNS algorithm on some mTRP instances that were addressed in Nucamendi-Guillén et al. (2016) and Angel-Bello et al. (2019). The main difference of mTRP and the WmTRP is that in the mTRP, the weight of all the nodes is equal to one. As part of the computational study of these articles, they tested their models and algorithms on 21 P-instances from the VRP literature (Augerat et al. 1995). While Angel-Bello et al. (2019) considered Euclidean distances that were rounded down to the nearest integer, Nucamendi-Guillén et al. (2016) considered Euclidean distances with up to two decimal points. The results of our computational experiments are given in Table 2. The “Instance” column gives the name of the instances, which include the number of nodes and repairmen in that instance. The column denoted by “Angel-Bello et al. (2019)” gives the extracted values from this article which are obtained for the mTRP using their mathematical model. In the next three columns, the results of testing the Saving+GVNS algorithm on these mTRP instances are given. Our algorithm found the optimal solutions to all of these instances with an average and maximum CRT of 4.84 and just over 11 s, respectively. We note that since Angel-Bello et al. (2019) used a mathematical model, they obtained the optimal solutions to all of these instances. In the next two columns denoted by Nucamendi-Guillén et al. (2016), we present the extracted results of their MIP model and metaheuristic algorithm labeled by IG+LS from their article. Their metaheuristic algorithm finds the optimal solutions to all the tested instances except for P-k8-n23. The results of testing the Saving+GVNS algorithm on these instances are given next. Similar to the case with integer distances, our algorithm found all the optimal solutions to these mTRP instances with an average CRT of under 5 s. While the Saving+GVNS was developed to solve the WmTRP, it is also capable of finding the optimal solutions to all of these mTRP instances verifying its robustness.

**Table 2:** Results of Saving+GVNS on mTRP instances

Instance	Angel-Bello et al. (2019)	Saving+GVNS			Nucamendi-Guillén et al. (2016)		Saving+GVNS		
		Best OFV	CRT	GAP (%)	Exact	IG+LS	Best OFV	CRT	GAP (%)
P-k8-n16	377	377	0.50	0	382.9	382.9	382.9	0.50	0
P-k2-n19	780	780	0.85	0	812.15	812.15	812.15	0.87	0
P-k2-n20	869	869	1.01	0	905.19	905.19	905.19	0.95	0
P-k2-n21	906	906	1.14	0	937.1	937.1	937.1	1.11	0
P-k2-n22	961	961	1.28	0	993.1	993.1	993.1	1.29	0
P-k8-n22	610	610	0.91	0	623.4	623.4	623.4	0.93	0
P-k8-n23	549	549	0.99	0	561.33	564.31	561.33	1.03	0
P-k5-n40	1491	1491	3.89	0	1537.79	1537.79	1537.79	3.77	0
P-k5-n45	1857	1857	5.25	0	1912.31	1912.31	1912.31	5.50	0
P-k7-n50	1493	1493	6.01	0	1547.89	1547.89	1547.89	5.71	0
P-k8-n50	1399	1399	5.56	0	1448.92	1448.92	1448.92	5.39	0
P-k10-n50	1256	1256	5.22	0	1296.48	1296.48	1296.48	5.47	0
P-k10-n51	1372	1372	5.50	0	1419.43	1419.43	1419.43	5.91	0
P-k7-n55	1705	1705	7.35	0	1766.56	1766.56	1766.56	7.31	0
P-k8-n55	1560	1560	7.24	0	1614.61	1614.61	1614.61	6.87	0
P-k10-n55	1395	1395	6.58	0	1438.6	1438.6	1438.6	6.76	0
P-k15-n55	1243	1243	6.50	0	1280.92	1280.92	1280.92	6.42	0
P-k10-n60	1622	1622	7.82	0	1676.35	1676.35	1676.35	8.15	0
P-k15-n60	1416	1416	7.70	0	–	–	1462.5	7.62	–
P-k10-n65	1866	1866	9.37	0	1928.46	1928.46	1928.46	9.98	0
P-k10-n70	2027	2027	11.02	0	2097.17	2097.17	2097.17	11.50	0
Average			4.84	0				4.91	0

### WmTRP instances

Next, the Saving+GVNS algorithm and our models are tested on WmTRP instances. This section is divided into two subsections based on the size of the networks and the number of customers in the solved instances.

#### Small instances

**Fig. 16:**
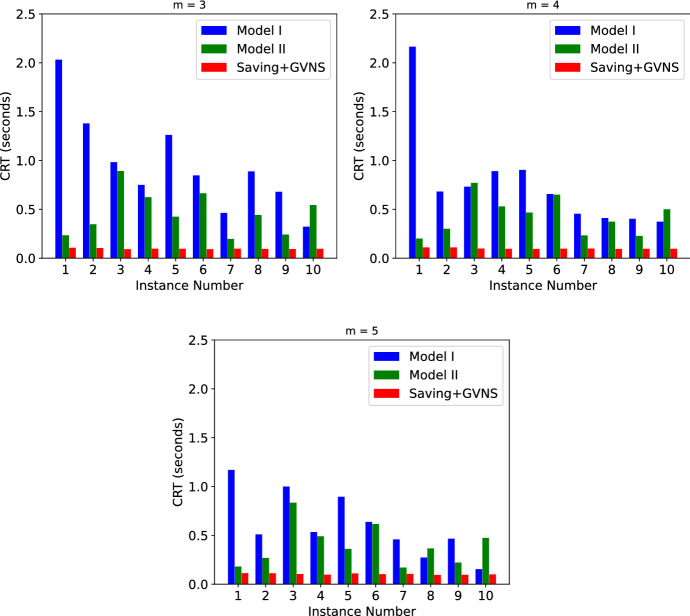
Results of the small instances with 10 patients

**Fig. 17:**
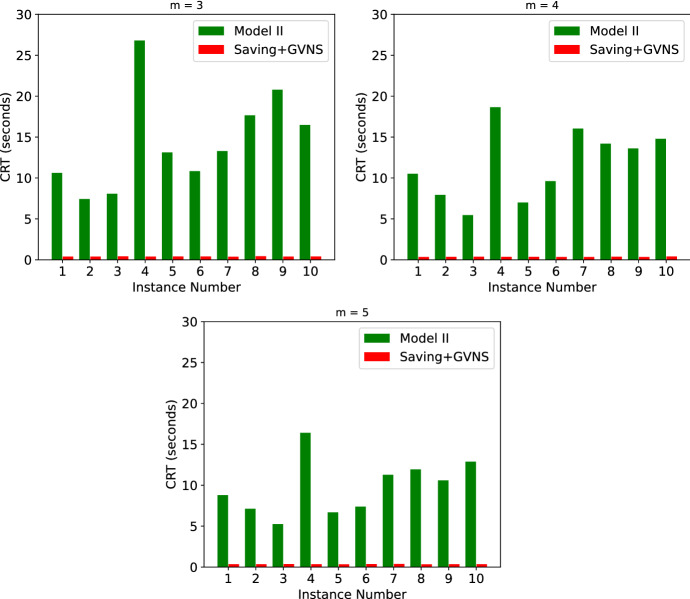
Results of the small instances with 20 patients

In order to provide a performance comparison of Models I and II, we adopted some small instances from the TRP literature (Salehipour et al. 2011). These small data sets have 10 and 20 nodes. Since these instances are not weighted, based on triage levels that are integer values between 1 and 5, we assigned a uniformly distributed number between 1 and 5 to each of the patient nodes as their weights and then tested both Models I and II and the Saving+GVNS algorithm with 3, 4 and 5 HSPTs on all the instances. The results of testing these instances with 10 and 20 nodes are given in Figs. 16 and 17, respectively.

For the instances with 10 nodes, both Models I and II found the optimal solutions in less than 2.5 s in all the instances. The Saving+GVNS algorithm was able to find the same optimal solutions in less than 0.2 s. As a result, Fig. 16 only shows the comparison of the CPU run time between these models and the algorithm. For the case with 20 patients however, Model I was not able to find any of the optimal solutions in a time limit of 1 h. As a result of this, we only gave the CRT comparison between Model II and Saving+GVNS in Fig. 17.

Among the instances with 20 patients, Model II found the optimal solution to all these instances with a maximum CRT of 26.79 s. An observation is that, when the number of HSPTs increases, on the same instance, the problem becomes computationally easier. For example, in instances with 20 nodes, the average CRT over the ten tested instances with 3 HSPTs is 14.51 s, while it reduces to 11.78 and 9.83 s with 4 and 5 HSPTs, respectively. As expected, adding more HSPTs on the same instances reduces the objective function value. While the average objective function value over ten instances with 20 nodes and 3 HSPTs is 3872.9, it reduces to 3408.4 and 3170.5 for 4 and 5 HSPTs, respectively. In both cases with 10 and 20 nodes, the Saving+GVNS algorithm performed found the optimal solution in all the cases. Since the algorithm was able to find the optimal solutions in all the 10 randomized runs for all the instances, the Best OFV and the Avg OFV are the same for all the small instances in this section.

#### Moderate-sized instances

In this section, we test the performance of the Saving+GVNS and Model II on moderate-sized instances. Some of these instances are directly taken from the literature and some are generated on instances adopted from the VRP literature.

##### Performance of the Saving+GVNS on WmTRP instances

Recently, Muritiba et al. (2021) introduced the WmTRP and proposed a branch-and-cut (B&C) algorithm to solve this problem. In their study, they addressed the application of the WmTRP for maintenance of speed cameras and introduced six categories of data sets denoted by “brd14051,” “d15112,” “d18512,” “fn14461,” “nrw1379” and “pr1002.” For each of these categories, they generated instances with 30, 40 and 50 nodes and assigned a floating point weight value between 0 and 2 to each of the nodes. For instances with 30 nodes, they used 6 repairmen and for instances with 40 and 50 nodes they used 8 and 10 repairmen, respectively. We note that in our context, these repairmen represent HSPTs and each node represents a patient location. Moreover, for each category and combination of number of nodes and repairmen, they generated 10 instances and reported the average objective function value obtained from their B&C algorithm over them.

**Fig. 18:**
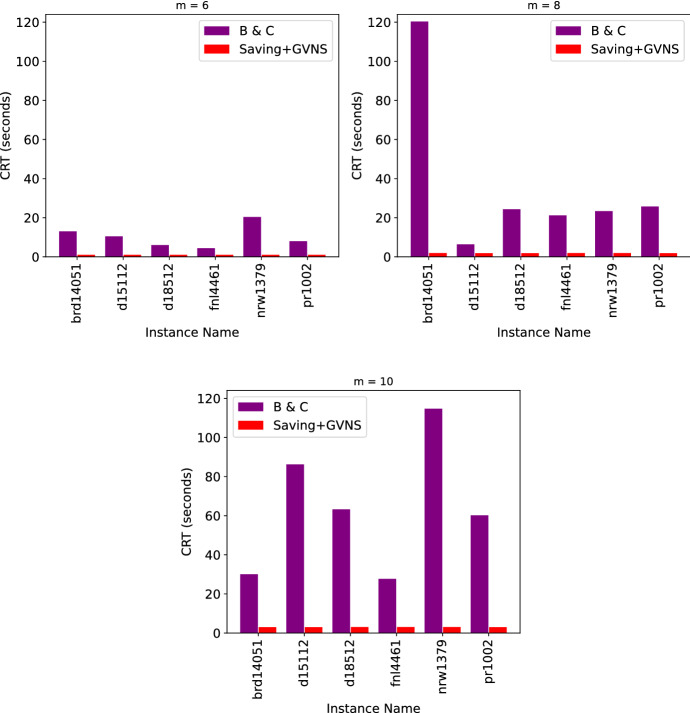
Results of the WmTRP instances from Muritiba et al. (2021)

The B&C algorithm developed by Muritiba et al. (2021) was able to find the optimal solution in all of their tested instances. Our Saving+GVNS algorithm was also able to find all the same optimal solutions in all the tested instances. Therefore, we only presented the CPU run times in Fig. 18. In this figure, we used the same run times that were presented in Muritiba et al. (2021). For their computational experiments, they coded their B&C algorithm in Java 8 and executed it on an Intel i7-3820 processor with eight cores running at 3.60 GHz and with 16 GB of random access memory, operating under Ubuntu 18.04. As an MILP solver, they used Gurobi 8.0. We can see that the Saving+GVNS algorithm was able to find the optimal solutions in a considerably shorter time.

Since Model II only handles integer weights, we have modified the instances described above and generated triage levels for each of the nodes (patients) using integer values between 1 and 5 and solved the new instances using both Model II and our Saving+GVNS algorithm. The results of these experiments are presented in Table 3. Since in Muritiba et al. (2021), the allocated weights to the nodes are floating points between 0 and 2, we modified them to integer weights using $w_i = \lceil \dfrac{w'_i}{0.4}\rceil$ formula where $w'_i$ shows the weights used by Muritiba et al. (2021) and $w_i$ denotes the modified weights that we used in our computational experiments. In order to obtain the optimality gaps of the Saving+GVNS algorithm presented in this table, we calculated them as: $\dfrac{{\textit{Best OFV}} - {\textit{Optimal Solution}}}{{\textit{Optimal Solution}}} \times 100\%$. We use the same formula to calculate the optimality gap throughout the article. This term is also referred to as the denominated relative percentage deviation (RPD) in the literature (Vallada et al. 2019; Abreu et al. 2020; Ying and Lin 2022). As it can be observed in Table 3, all the instances were solved optimally using Model II within a highest CPU time of 20 min. Furthermore, our Saving+GVNS algorithm was able to find the similar optimal solution to all the instances in an average run time of just over 2 s.

**Table 3:** Results of modified WmTRP instances from Muritiba et al. (2021)

Instance	*n*	*m*	Model II		Saving+GVNS		
			Optimal OFV	CRT	Best OFV	CRT	Opt GAP (%)
brd14051	30	6	310,651.72	110.23	310,651.72	1.10	0.00
d15112	30	6	823,829.90	113.33	823,829.90	1.11	0.00
d18512	30	6	308,056.26	117.30	308,056.26	1.12	0.00
fnl4461	30	6	176,340.73	100.76	176,340.73	1.09	0.00
nrw1379	30	6	108,902.86	105.30	108,902.86	1.09	0.00
pr1002	30	6	589,241.26	74.68	589,241.26	1.12	0.00
brd14051	40	8	366,822.74	393.22	366,822.74	2.07	0.00
d15112	40	8	1,041,017.05	383.81	1,041,017.05	2.04	0.00
d18512	40	8	401,214.84	424.15	401,214.84	2.05	0.00
fnl4461	40	8	223,372.23	390.99	223,372.23	2.06	0.00
nrw1379	40	8	131,035.45	381.57	131,035.45	2.08	0.00
pr1002	40	8	772,348.63	365.50	772,348.63	2.04	0.00
brd14051	50	10	459,627.24	1055.44	459,627.24	3.25	0.00
d15112	50	10	1,289,289.21	1020.96	1,289,289.21	3.30	0.00
d18512	50	10	485,326.37	1132.05	485,326.37	3.24	0.00
fnl4461	50	10	281,773.14	1093.28	281,773.14	3.27	0.00
nrw1379	50	10	165,271.23	1063.27	165,271.23	3.22	0.00
pr1002	50	10	961,273.67	969.01	961,273.67	3.29	0.00
Average			494,188.58	516.38	494,188.58	2.14	0.00

##### VRP instances

In this section, we further analyze the performance of Model II and verify the performance of the Saving+GVNS algorithm. Since Model I was not capable of solving instances with 20 nodes within 1 h time limit, we did not test its performance in this section for the moderate-sized instances. Instead, we adopted 55 instances from the VRP literature (Augerat et al. 1995) with up to 60 nodes and 15 vehicles. We have adopted these instances from three sets of instances denoted by A, B and P in this study. Each instance is named as X-kY-nZ where X denotes whether that instance is from set A, B, or P, Y gives the number of vehicles in that instance and Z gives the number of nodes in the VRP problem. For each of these instances, we used the same number of HSPTs with the number of vehicles in the VRP problem. Furthermore, since these instances are not weighted, we tested them under two different scenarios. In the first scenario, we assigned weights to each node such that the summation of the weights equals 100. This could for example represent triage values under a different scale or a case where importance percentage is allocated to the nodes (patients). In the second scenario, similar to previous parts, we assigned triage levels using uniformly distributed integer values between 1 and 5 for each of the patients (nodes). The results of these experiments for the instances denoted by A, B and P are given in Tables 4, 5 and 6. For each instance, under each scenario, we report the optimal objective function value and the CPU run time (in seconds) of Model II and denoted them by Optimal OFV and CRT, respectively. For each instance, we also give the results of the Saving+GVNS algorithm including the best objective function value (Best OFV), the CPU run time in seconds and the optimality gap calculated similar to what was explained above.

Table 4 gives the results of the experiments on the A-instances. In these instances, the summation of the triage levels for all the patients is assumed to be 100. For the second scenario, however, since the triage level or weight of each patient is a uniformly generated random integer between 1 and 5, the summation of the triage levels over all the patients is variable and hence is given as column $\mathcal {W}$ in the tables. As it can be observed in Table 4, Model II solved all the instances of both scenarios optimally, with a maximum CRT of 1302.68 for the A-k9-n55 instance in the second scenario. The maximum CRT of the first scenario belongs to A-k7-n57 with 231.57 s. As it was expected, once the number of nodes and/or the summation of the triage levels over all the patients increases, the CRT of the Model II also increases. This is evident when comparing the same instances over the first and second scenarios. Given that $\mathcal {W}$ is fixed at 100 for all the instances in the first scenario, in those second scenario instances that $\mathcal {W}$ is more 100, generally, the CPU run time is higher than the first scenario and vice versa. But a comparison between the CRT of the instances shows that in general, the same sized instances with less HSPTs are more difficult to solve. For example, this can be observed comparing instances A-k5-n39 and A-k6-n39. The results given in this table shows the good performance of the Saving+GVNS algorithm. Our metaheuristic algorithm found the optimal solution in all the tested instances and in the largest instance, i.e., A-k9-n60, the optimal solutions of the first and second scenarios were found in 8.23 and 8.10 s, respectively. We note that development of Model II enabled us to find the optimal solutions in these instances which were used to test the performance of the Saving+GVNS algorithm. We recall that the Best OFV values reported in Table 4 are extracted from 10 runs of our Saving+GVNS algorithm over each instance. In some of the instances, the optimal solution was not found in all the 10 runs (i.e., Best OFV is not equal to the Avg OFV). However, the average gap of all the tested A-instances between the Best OFV and the Avg OFV of both scenarios is under 0.01%. Among different instances of the first scenario, instance A-k5-n39 had the highest gap between the Avg OVF and the Best OFV with 0.18%. In the second scenario, instance A-k9-n55 had the largest gap between Avg OFV and Best OFV with 0.08%.

**Table 4:** Results of the A-instances

Instance	Scenario 1					Scenario 2					
	Model II		Saving+GVNS			Model II			Saving+GVNS		
	Optimal OFV	CRT	Best OFV	CRT	Opt Gap (%)	$\mathcal {W}$	Optimal OFV	CRT	Best OFV	CRT	Opt Gap (%)
A-k5-n32	7296.40	99.91	7296.40	2.34	0	103	7035.51	241.48	7035.51	2.29	0
A-k5-n33	4954.27	108.75	4954.27	2.27	0	94	5130.66	95.91	5130.66	2.30	0
A-k6-n33	4781.73	99.96	4781.73	2.35	0	89	4070.04	42.23	4070.04	2.38	0
A-k5-n34	5987.11	93.97	5987.11	2.65	0	99	6044.87	120.61	6044.87	2.46	0
A-k5-n36	6806.18	114.30	6806.18	2.96	0	113	7410.94	198.52	7410.94	2.78	0
A-k5-n37	5292.81	111.20	5292.81	3.43	0	113	6006.35	198.99	6006.35	3.28	0
A-k6-n37	6596.17	108.36	6596.17	3.01	0	103	6703.96	67.80	6703.96	3.04	0
A-k5-n38	5173.79	170.51	5173.79	3.29	0	106	5463.88	155.87	5463.88	3.23	0
A-k5-n39	5637.15	120.08	5637.15	3.76	0	109	6075.69	154.98	6075.69	3.51	0
A-k6-n39	4488.34	102.75	4488.34	3.45	0	110	5921.42	155.14	5921.42	3.21	0
A-k7-n44	4888.18	123.52	4888.18	4.25	0	113	6297.61	248.55	6297.61	4.03	0
A-k6-n45	5739.56	124.03	5739.56	5.02	0	139	8087.72	387.15	8087.72	4.91	0
A-k7-n45	6067.98	206.35	6067.98	4.67	0	127	8121.10	636.12	8121.10	4.49	0
A-k7-n46	4556.78	145.36	4556.78	4.81	0	132	6725.65	403.29	6725.65	4.59	0
A-k7-n48	6321.02	188.89	6321.02	5.39	0	135	8318.74	469.96	8318.74	5.33	0
A-k7-n53	5975.00	189.28	5975.00	6.58	0	147	8126.08	768.23	8126.08	6.60	0
A-k7-n54	6344.95	231.57	6344.95	7.01	0	161	9566.45	1031.48	9566.45	6.75	0
A-k9-n55	4674.91	159.46	4674.91	6.89	0	173	7875.65	1302.68	7875.65	6.82	0
A-k9-n60	6113.35	207.74	6113.35	8.23	0	160	8789.17	1017.48	8789.17	8.10	0

Table 5 gives the results of testing Model II and the Saving+GVNS algorithm on B-instances. The columns of this table are the same as those in Table 4. We can see that all the instances were solved optimally using Model II within the 3 h time limit. For instances B-k7-n52 and B-k7-n56 in the second scenario, the CRT of Model II increased to more than 2 h to find the optimal solutions. However, except these two instances and B-k7-n57 of the second scenario, all the remaining instances were solved optimally within 1 h. Among these three instances, B-k7-n52 was solved in less than 62 min. The performance of the Saving+GVNS algorithm is impressive on the B-instances as well. Our metaheuristic algorithm found the optimal solution to all the tested instances with a maximum CRT of just over 8 s for the B-k7-n57 instances. The optimal solutions of the remaining instances were found using our algorithm in less than 8 s. The significant difference between the CRT of Model II and the Saving+GVNS algorithm combined with the fact that our algorithm found the optimal solutions in all of these instances verifies the superior performance of the Saving+GVNS algorithm. In some of the B-instances, not all the 10 runs were able to find the optimal solutions (i.e., Best OFV was not equal to Avg OFV). In the instances of the first and second scenarios, instances B-k7-n50 and B-k6-n43 had the highest gap between Best OFV and Avg OFV with 0.69 and 0.15%, respectively. The average of this gap over all the B-instances remained under 0.05% for the first scenario and under 0.03% for the second scenario.

**Table 5:** Results of the B-instances

Instance	Scenario 1					Scenario 2					
	Model II		Saving+GVNS			Model II			Saving+GVNS		
	Optimal OFV	CRT	Best OFV	CRT	Opt Gap (%)	$\mathcal {W}$	Optimal OFV	CRT	Best OFV	CRT	Opt Gap (%)
B-k5-n31	6036.14	166.60	6036.14	2.08	0	78	4802.60	86.20	4802.60	1.95	0
B-k5-n34	6810.04	226.79	6810.04	2.75	0	112	7378.63	449.60	7378.63	2.67	0
B-k5-n35	8581.57	229.37	8581.57	2.77	0	110	8841.24	290.26	8841.24	2.71	0
B-k6-n38	5482.06	186.67	5482.06	3.11	0	114	6401.15	233.54	6401.15	3.06	0
B-k5-n39	4579.46	170.70	4579.46	3.91	0	120	5566.80	463.95	5566.80	3.86	0
B-k6-n41	5605.00	182.23	5605.00	3.97	0	114	6232.10	313.98	6232.10	3.81	0
B-k6-n43	4715.92	208.99	4715.92	4.22	0	134	6139.92	585.43	6139.92	4.31	0
B-k7-n44	5175.44	194.82	5175.44	4.22	0	132	7082.53	566.95	7082.53	4.56	0
B-k5-n45	5173.07	269.98	5173.07	5.54	0	127	6596.38	392.02	6596.38	5.19	0
B-k6-n45	4647.59	280.10	4647.59	4.86	0	129	5642.13	798.00	5642.13	5.19	0
B-k7-n50	4363.89	1148.75	4363.89	5.82	0	145	6264.68	769.08	6264.68	5.72	0
B-k8-n50	5616.58	190.32	5616.58	5.67	0	138	7864.20	938.85	7864.20	5.59	0
B-k7-n51	5907.48	911.26	5907.48	6.01	0	168	10,011.65	2547.48	10,011.65	5.99	0
B-k7-n52	4945.94	790.19	4945.94	6.60	0	173	8782.00	3699.47	8782.00	6.42	0
B-k7-n56	4063.03	559.67	4063.03	8.29	0	188	8116.65	8507.35	8116.65	7.56	0
B-k7-n57	6423.37	1587.16	6423.37	8.45	0	173	11,483.92	10,467.23	11,483.92	8.65	0
B-k9-n57	7816.18	294.93	7816.18	7.35	0	165	12,974.29	1569.00	12,974.29	7.67	0

Table 6 gives the results of Model II and the Saving+GVNS algorithm on P-instances. The columns of this table are the same as those of Table 4. The results of the P-instances are similar to those of the A and B instances. Impressively, in all the tested instances, the Saving+GVNS found the optimal solution in less than 8 s. Model II also found the optimal solution to all the tested instances within the given 3 h time limit. Similar to A and B instances, the optimal solution was not found in some runs of a few instances. For the first scenario, the maximum gap between Best OFV and Avg OFV was in instance P-k10-n51 with 0.22% and the average of these gaps was 0.04%. For the second scenario, the average gap between Best OFV and Avg OFV among all the P-instances remained under 0.06% and the maximum gap occurred in instance P-k5-n40 with 0.79%.

**Table 6:** Results of the P-instances

Instance	Scenario 1					Scenario 2					
	Model II		Saving+GVNS			Model II			Saving+GVNS		
	Optimal OFV	CRT	Best OFV	CRT	Opt Gap (%)	$\mathcal {W}$	Optimal OFV	CRT	Best OFV	CRT	Opt Gap (%)
P-k8-n16	2395.74	29.19	2395.74	0.47	0	39	1004.64	1.44	1004.64	0.48	0
P-k2-n19	4011.38	66.22	4011.38	0.85	0	62	2735.18	19.89	2735.18	0.82	0
P-k2-n20	3867.48	50.29	3867.48	0.95	0	57	2462.62	19.07	2462.62	0.95	0
P-k2-n21	4339.84	78.24	4339.84	1.15	0	52	2245.47	9.49	2245.47	1.08	0
P-k2-n22	3940.58	66.67	3940.58	1.21	0	65	3015.94	32.34	3015.94	1.23	0
P-k8-n22	3053.37	47.30	3053.37	0.92	0	67	2094.43	13.28	2094.43	0.92	0
P-k8-n23	2665.63	49.02	2665.63	1.02	0	65	1606.01	11.61	1606.01	1.02	0
P-k5-n40	3674.48	138.55	3674.48	3.74	0	107	4186.52	112.35	4186.52	3.65	0
P-k5-n45	3910.11	187.30	3910.11	5.89	0	147	5865.40	904.65	5865.40	5.14	0
P-k10-n50	2728.15	108.51	2728.15	5.33	0	146	3711.14	445.80	3711.14	5.59	0
P-k7-n50	3155.27	131.53	3155.27	5.63	0	149	4542.04	510.07	4542.04	5.58	0
P-k8-n50	2917.60	124.52	2917.60	5.48	0	139	4336.72	453.26	4336.72	5.63	0
P-k10-n51	2871.04	111.48	2871.04	5.66	0	153	4228.13	517.90	4228.13	5.42	0
P-k10-n55	2752.35	114.73	2752.35	6.59	0	153	4253.81	547.59	4253.81	6.28	0
P-k15-n55	2335.54	95.41	2335.54	6.22	0	174	4160.29	825.94	4160.29	6.11	0
P-k7-n55	2956.70	137.60	2956.70	7.33	0	171	5246.30	994.17	5246.30	7.38	0
P-k8-n55	2819.32	118.55	2819.32	6.64	0	127	3763.03	309.54	3763.03	6.74	0
P-k10-n60	2583.20	229.74	2583.20	7.93	0	184	4948.53	1212.28	4948.53	7.70	0
P-k15-n60	2317.83	99.67	2317.83	7.44	0	184	4535.49	1041.99	4535.49	7.51	0

## Case study

**Fig. 19:**
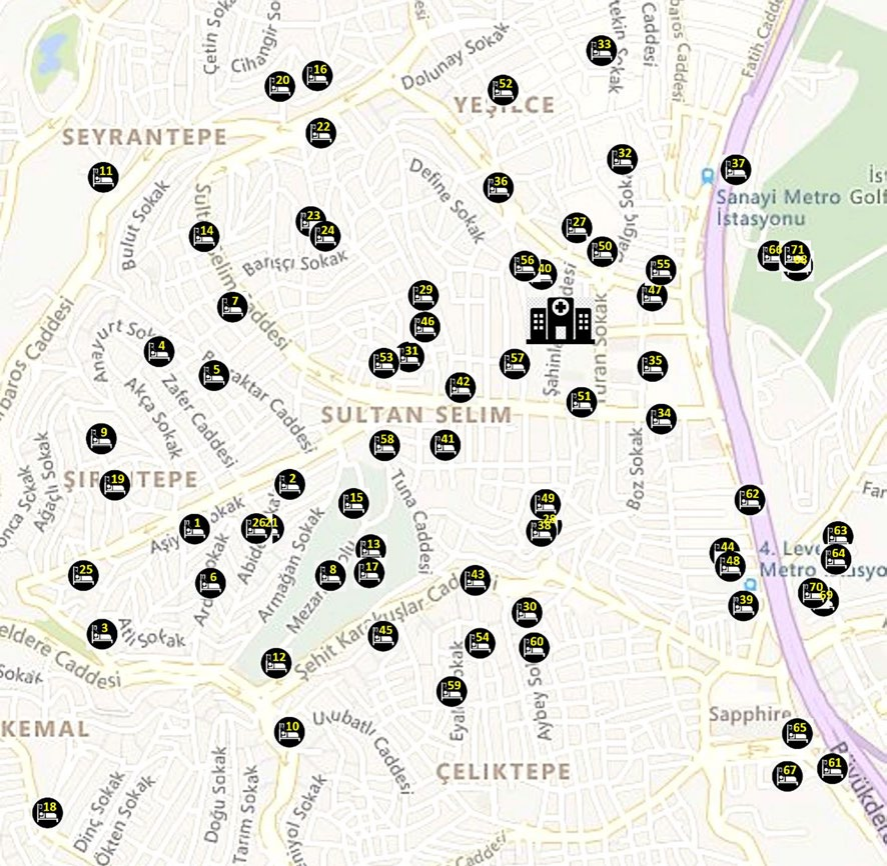
The geographical locations of the hospital and patients

**Fig. 20:**
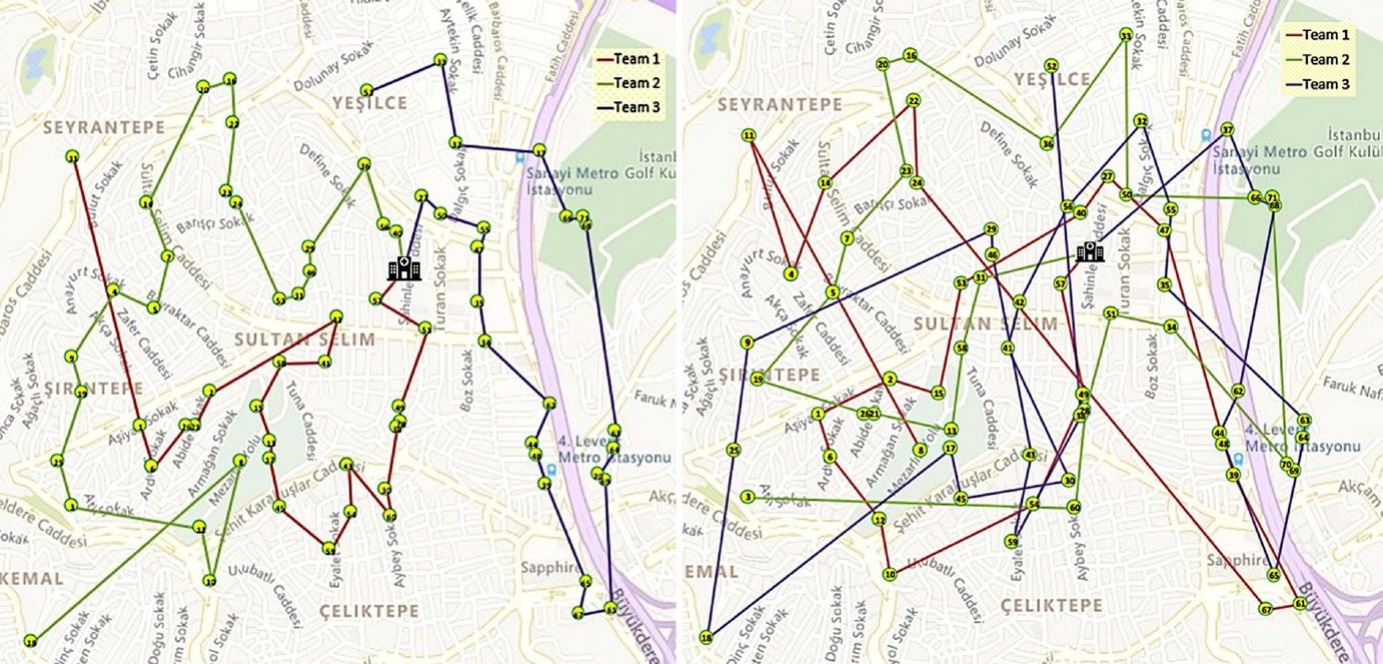
Visualization comparison of weighted and non-weighted objectives

In this section, we provide a case study of our problem related to the filiation operations during the pandemic for the patients residing in the Kağıthane district of the European side of Istanbul. The data consists of 647 patients who are assigned to clusters according to their home addresses, that is, their geographical proximity to each other. The clusters are formed such that each cluster has about the same number of patients and the sum of the distances between the patients in each cluster is minimized by means of a clustering algorithm. Using this clustering method, the 647 patients are clustered into 9 regions. In addition, a priority score based on the triage level which is an integer number between 1 and 5 is assigned to each patient, indicating the urgency of visiting that patient. The available HSPTs (which contain one or more personnel such as a doctor, nurse or other caregivers) are assigned to these regions such that their workloads are balanced and patients that are geographically close to each other are assigned to the same HSPT. Among these nine regions, we selected region 2 with 71 patients and three HSPTs to provide the visualization of the placement of the patients in Figs. 19 and the obtained routes from the Saving+GVNS algorithm for the three HSPTs on the right side of Fig. 20. Here, we note that the traversal time between two coordinates was calculated using the Haversine formula considering an average speed of 30 km/h. For each patient, a service time of 15 min (0.25 h) is considered as their service time. The weighted latency objective function is calculated based on hours each patient have to weight until they are initially met with a HSPT. For the tested instances in this section, we applied our Saving+GVNS algorithm for ten repetitions and reported the best results among them.

The solution obtained by our Saving+GVNS algorithm is depicted on the right side of Fig. 20. The objective function value for visiting these 71 patients with three HSPTs is 512.91. The objective function value is calculated as the total weighted latency of the patients, where the latency of the patients is calculated based on hours. The total traveled distance according to the obtained routes is 16.64 km and the total time including the service times is 18.30 h (i.e., 6.10 h per team on average). As can be observed on the right side of Fig. 20, with a weighted latency objective, the obtained routes are visually different from typical efficient solutions for routing problems. To further illustrate this, on the left side of Fig. 20, we gave the illustration of the routes when the weights are ignored, i.e., are set to 1 for all the patients. Although in this solution the total traveled distance reduces to 14.18 km, the corresponding weighted latency objective for these routes is 695.16 which is 35.53% higher than the 512.91 found using a weighted latency objective.

To investigate the impact of the number of HSPTs on the solutions, we solved the region 2 instance using $m=1,2,4$ and 5 HSPTs as well. The results of these experiments are given in Table 7. In this table, columns “*n*” and “*m*” show the number of patients and the number of HSPTs. Columns “OFV”, “CRT (s)”, “TD (km)”, “TT (hr)” and “TT (hr) per team” indicate the objective function value, CPU run time in seconds, total traveled distances, total time (travel and service) and the average total time per team, respectively. According to the obtained results, as expected, once the number of HSPTs increases, the obtained objective function value decreases. We also observe that the rate of this deduction decreases when more HSPTs are considered. For instance, while this deduction is around 34% when instead of one HSPT, two HSPTs are utilized, this deduction is under 18% when the number of HSPTs increases from four to five. Another important observation is based on the last column. While with only one HSPT the tasks will be finished after 18.26 h, when 5 HSPTs are used, the average time of each team decreases to 3.67 h.

**Table 7:** Results of region 2 with different number of HSPTs

Instance	*n*	*m*	OFV	CRT (s)	TD (Km)	TT (hr)	TT (hr) per team
Region 2	71	1	1145.98	95.34	15.36	18.26	18.26
		2	756.69	52.54	13.51	18.20	9.10
		3	512.91	25.90	16.64	18.30	6.10
		4	365.85	20.97	17.29	18.33	4.58
		5	301.72	12.34	18.22	18.36	3.67

**Fig. 21:**
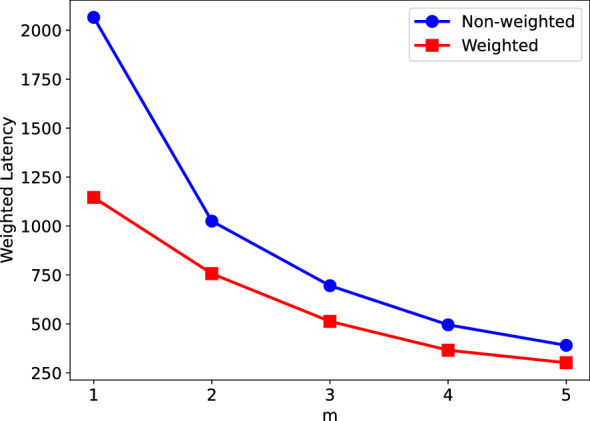
Comparison of the obtained routes when the weights are ignored

In order to investigate the impact of considering patient priorities, we also solved the same instances by considering equal weights for all the patients. In this scenario, we ignored the weights and set the triage levels of all patients equal to 1. This is equivalent to a “un-weighted” problem. We then solved the instance of the second region using different number of HSPTs, i.e., $m=1,2,3,4,$ and 5. The results are provided in Fig. 21, in which a comparison of the OFV of the un-weighted problem with the original problem (solution provided in Table 7) is given. To be consistent in comparing the results, for the un-weighted cases, we have extracted the routes for each of the HSPTs and calculated the adjusted objective function value using the corresponding triage level for each of the patients. While using the un-weighted latency objective, the routes might be shorter (as depicted in Fig. 21), ignoring the triage level in planning the routes can result in finding inefficient solutions when a weighted latency objective is considered. When the total weighted latency is calculated for these routes, it is seen that the solutions are on average 43.21% worse than the solutions found directly from the weighted objective function.

**Fig. 22:**
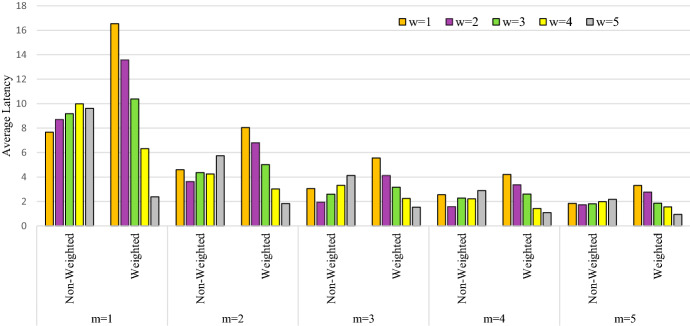
Latency of patient categories based on the objective

To further analyze the importance of incorporating the triage levels, we provide the average latency (in hours) of patients categorized by triage levels and the number of teams in Table 8. For example, the average latency of patients with $w=1$ and $m=1$ is 7.68 and 16.54 h in non-weighted and weighted cases, respectively. The results are given in Fig. 22. As it can be observed, for the un-weighted problem, the average latency of the more urgent patients having a triage level of 5 is consistently more than the average latency of the same patients category under a weighted objective. This verifies the importance of considering the triage levels of the patients in this home healthcare delivery problem. However, it can be observed that, in the weighted version, the latency of the patient categories with lower triage levels can be considerably more than the same patients in the un-weighted scenario. For example, when $m=1$, the latency of the least urgent patients with $w=1$, is more than twice in the weighted version when compared to the un-weighted solution.

**Table 8:** Average latency (in hours) for different patient categories with weighted and un-weighted objectives

Weight	$m=1$		$m=2$		$m=3$		$m=4$		$m=5$	
	Non-weighted	Weighted	Non-weighted	Weighted	Non-weighted	Weighted	Non-weighted	Weighted	Non-weighted	Weighted
$w=1$	7.68	16.54	4.59	8.04	3.05	5.56	2.55	4.21	1.84	3.31
$w=2$	8.71	13.58	3.62	6.80	1.94	4.13	1.58	3.36	1.72	2.76
$w=3$	9.18	10.38	4.36	5.01	2.59	3.16	2.27	2.61	1.81	1.86
$w=4$	9.99	6.32	4.25	3.03	3.32	2.25	2.21	1.42	1.98	1.55
$w=5$	9.62	2.38	5.73	1.83	4.12	1.53	2.89	1.08	2.17	0.93

As mentioned earlier, we selected region 2 among the nine regions to provide its visualization and further analysis. In this part, we present the results on the remaining eight regions using three HSPTs for each of the regions. For each of these regions, a hospital is considered as the depot and hence, for each of these regions, we have a single-depot problem. Moreover, in order to see the effect of a centralized system and to highlight the capability of our proposed GVNS in solving the multi-depot case, we solve the multi-depot centralized case with all patients and the nine depots (hospitals). Similar to the case for each region where three teams were considered, in the multi-depot case we assume three HSPTs are pre-positioned in each of the depots (i.e., in total there are 27 teams). We refer to the first case as “decentralized” and the latter as “centralized.” Table 9 provides the results for each of these cases. In this table, the “Region” column indicates the selected region, and the remaining columns are translated in the same way as Table 7. The last row shows the improvements in the centralized cases compared to the decentralized ones. The total objective function value in the decentralized case is 4524.58, whereas in the centralized case it reduces to 4398.96 (a 2.78% improvement). We can also see that solving the centralized instance with 646 patients took 36.48% less time compared to the summation of the times for the decentralized cases over the nine regions. This analysis shows that considering a centralized system can improve patients’ satisfaction and decrease total latency.

**Table 9:** Comparison of centralized and decentralized models

	Region	*n*	*m*	OFV	CRT (s)	TD (Km)	TT( hr)	TT (hr) per team
Decentralized	1	71	3	474.48	34.41	19.88	18.41	6.14
	2	71	3	512.91	25.90	16.64	18.30	6.10
	3	72	3	588.65	29.72	17.38	18.58	6.19
	4	72	3	463.96	29.96	14.72	18.49	6.16
	5	72	3	512.42	32.35	17.62	18.59	6.20
	6	72	3	453.58	31.31	14.29	18.48	6.16
	7	72	3	542.26	30.29	18.22	18.61	6.20
	8	72	3	466.30	28.38	14.91	18.50	6.17
	9	72	3	510.02	32.30	17.01	18.57	6.19
Total sum		646		4524.58	274.61	150.67	166.53	55.51
Centralized	Joint regions	646	$(9*3)=27$	4398.96	174.44	142.76	166.26	55.42
Improvement				2.78%	36.48%	5.25%	0.16%	0.16%

## Conclusion

We considered a real-life emergency situation such as the COVID-19 outbreak, where standard home healthcare services such as testing or filiation must be provided to the patients at their homes. In this context, we investigated a problem where multiple home healthcare service provider teams (HSPTs) should provide service to a given set of patients. The patients are prioritized by triage levels based on the severity of their condition or the urgency of servicing them. Each patient should be serviced by exactly one HSPT and the objective is to minimize the summation of the weighted times at which the patients are serviced. We introduced a number of valid constraints for a level-based model driven by a transformation of the input graph and developed an efficient exact model for this problem which is capable of solving moderate-sized instances. We then developed a metaheuristic (Saving+GVNS) algorithm to solve larger instances in shorter time. In our metaheuristic algorithm, the initial solutions are constructed by a problem-specific saving procedure and then improved by a GVNS algorithm.

We performed extensive computational tests. Initially, to compare our Saving+GVNS algorithm with best known algorithms from the literature, we tested its performance on two well-studied special cases of our problem. We then tested our models and the Saving+GVNS algorithm on standard traveling repairman problem and as well as and the vehicle routing problem test instances. To conduct these experiments, we first verified the importance of developing our level-based model to solve even small-sized instances by comparing its performance with a standard MIP model on small-sized instances. We then tested the performance of the Saving+GVNS algorithm by comparing its results with those from a recent article that addresses the weighted K-traveling repairman problem (Muritiba et al. 2021). On these instances, our algorithm was able to find, impressively, all the optimal solutions within merely 2 s.

In order to show the good performance of the Saving+GVNS algorithm, we next compared its results with the level-based model on moderate-sized instances. Our level-based model was able to solve all the moderate-sized instances within a 3 h time limit. Our algorithm was able to find similar optimal solutions to all the small and moderate-sized instances within a maximum CPU run time of 9 s. We also provided a detailed analysis of a real-life case study, based on a data set of a district in the European side of the Istanbul city, which consists of 647 patients. Finally, in order to further analyze the performance of our metaheuristic algorithm, we tested instances with up to 500 nodes and 20 teams and reported the results in Appendix A.

Analyzing the extension of the studied problem where the triage levels of the patients are not known in advance and are only revealed online can be further analyzed. In such cases, not only the triage level will be online, the service time will also be online and it can only be estimated after the conditions of a patient is examined by the HSPTs. Lastly, although we presented our problem and the solution approaches in the context of home health care services, and in particular our case study was related to the filiation services, they can be applied to cases with visits serving other purposes in other application areas, such as repair and maintenance services. The methods can be applied to solve routing and scheduling problems in other check-up services and even distribution problems in city logistics where having a service-oriented objective is important.

## Data Availability

In our study, we have used several data sets from the literature. We used TSPLib instances presented in Abeledo et al. (2013) and “P-instances” from Augerat et al. (1995) to show the effectiveness of our algorithm on TRP and mTRP instances. We used the instances from Salehipour et al. (2011) to test the performance of Model I, Model II and our metaheuristic algorithm. We used the instances from Muritiba et al. (2021) to show the effectiveness of our level-based IP model and metaheuristic on a similar problem recently introduced to the literature. We used, modified versions of “A”, “B” and “P” instances from Augerat et al. (1995). Finally, we developed our case study data set based on the Kağıthane district in Istanbul, Turkey. This data set includes 647 patients that are clustered into nine groups for meeting their demand. All these data sets are available to be shared with interested researchers upon their request.

## Appendix A: Large instances

In this section, we adopted and tested larger instance from the TRP literature (Salehipour et al. 2011) to further analyze the performance of our Saving+GVNS algorithm. These instances were initially generated and tested on the traditional TRP with 1 repairman considering 50, 100, 200 and 500 non-weighted nodes. In order to assign a weight to each of the nodes, which represent triage levels in our study, we generated and assigned a uniformly distributed random number between 1 and 5 to each node. The results of testing the Saving+GVNS algorithm on these instances are given in Tables 10 and 11.

In Table 10, the results of testing the Saving+GVNS algorithm on instances with 50 and 100 nodes are given. In this table, the left hand side columns give the results of the instances with 50 nodes and the columns on the right side give the results of the tested instances with 100 nodes. With both 50 and 100 nodes, 10 instances were introduced in (Salehipour et al. 2011). These instances are distinguished by $n-Ri$ notation, where *n* shows the number of nodes and *i* shows the instance number, $i \in \{1,\ldots ,10\}$. The name of each instance is given in Instance column. The number of HSPTs in each instance is given by *m*. While with 50 nodes, we tested the Saving+GVNS algorithm with 3, 4 and 5 HSPTs, with 100 nodes, we tested 5, 8 and 10 HSPTs for each instance. We provided the best objective function value and the average objective function value in the next columns. The CPU time of the Saving+GVNS algorithm on average are provided in column CRT. We also presented the average Saving+GVNS gap over the 10 runs of each instance in the Saving+GVNS Gap column. This is calculated as $\dfrac{{\textit{Avg OFV}} - {\textit{Best OFV}}}{{\textit{Best OFV}}} \times 100\%$.

**Table 10:** Large instances results for instances with 50 and 100 nodes

$n = 50$						$n = 100$					
Instance	*m*	Best OFV	Avg OFV	CRT	Saving+GVNS GAP (%)	Instance	*m*	Best OFV	Avg OFV	CRT	Saving+GVNS GAP (%)
50-R1	3	10,939	10,939.0	13.80	0.00	100-R1	5	25,076	25,268.1	28.24	0.77
	4	8830	8830.0	9.96	0.00		8	19,731	19,859.8	16.34	0.65
	5	7946	7946.0	8.12	0.00		10	18,336	18,416.1	14.17	0.44
50-R2	3	11,568	11,568.0	13.13	0.00	100-R2	5	22,946	23,153.1	31.60	0.90
	4	9645	9645.0	10.23	0.00		8	18,191	18,224.0	17.35	0.18
	5	8674	8704.0	8.74	0.35		10	16,914	16,949.1	13.62	0.21
50-R3	3	13,631	13,922.2	14.30	2.14	100-R3	5	23,815	23,897.4	28.31	0.35
	4	10,896	10,911.0	10.72	0.14		8	19,498	19,563.6	17.85	0.34
	5	9352	9452.0	7.85	1.07		10	18,281	18,344.9	15.06	0.35
50-R4	3	13,563	13,590.6	13.43	0.20	100-R4	5	26,636	26,726.7	31.61	0.34
	4	11,207	11,301.5	10.51	0.84		8	22,210	22,273.4	18.76	0.29
	5	10,033	10,038.9	8.36	0.06		10	21,014	21,046.0	14.56	0.15
50-R5	3	12,666	12,677.6	13.51	0.09	100-R5	5	23,286	23,316.9	32.58	0.13
	4	10,660	10,660.0	9.84	0.00		8	17,521	17,569.4	16.48	0.28
	5	9683	9689.5	8.57	0.07		10	16,187	16,235.2	22.89	0.30
50-R6	3	11,964	11,964.0	14.82	0.00	100-R6	5	24,859	25,178.5	30.63	1.29
	4	9813	9813.0	10.30	0.00		8	18,526	18,665.9	17.09	0.76
	5	8907	8919.0	8.29	0.13		10	16,826	16,912.9	13.19	0.52
50-R7	3	12,049	12,049.0	13.79	0.00	100-R7	5	25,890	26,074.4	28.74	0.71
	4	9606	9606.0	10.82	0.00		8	20,736	20,775.0	15.90	0.19
	5	8442	8442.0	8.95	0.00		10	19,366	19,431.2	14.67	0.34
50-R8	3	15,024	15,064.5	15.24	0.27	100-R8	5	20,010	20,038.5	28.31	0.14
	4	12,543	12,543.0	10.37	0.00		8	16,047	16,083.9	15.86	0.23
	5	11,548	11,567.0	8.31	0.16		10	14,812	14,848.6	13.51	0.25
50-R9	3	14,356	14,356.0	14.40	0.00	100-R9	5	20,729	21,078.1	27.80	1.68
	4	12,471	12,489.4	9.83	0.15		8	15,338	15,413.5	16.98	0.49
	5	11,283	11,321.6	8.24	0.34		10	13,628	13,760.9	14.57	0.98
50-R10	3	14,849	14,945.5	14.08	0.65	100-R10	5	19,278	19,489.2	31.25	1.10
	4	12,110	12,110.0	10.67	0.00		8	14,972	14,975.1	17.48	0.02
	5	10,748	10,756.2	8.36	0.08		10	13,680	13,740.5	14.14	0.44
Average				10.91	0.22	Average				20.65	0.49

As it can be observed, the average CRT of the instances with 50 nodes is 10.91 s and the average Saving+GVNS gap over these instances is 0.22%. The maximum Saving+GVNS gap over instances with 50 nodes is 2.14% for the 50-R3 instance with 3 HSPTs. These low Saving+GVNS gaps verify the robustness of our algorithm. The maximum reported CRT over the instances with 50 nodes is observed in the 50-R8 instance with 15.24 s. We see that the average CRT reduces as the number of HSPTs increases. In an example from instances with 3 HSPTs, the average CRT is 14.05 s, and it lowers to 10.33 and 8.38 s in instances with 4 and 5 HSPTs, respectively.

In the instances with 100 nodes, we set the number of HSPTs equal to 5, 8 and 10. Over these instances, the average CRT is 20.65 s and the average Saving+GVNS gap is 0.49%. As expected, compared to the case with 50 nodes, when the number of nodes increases, both the average CRT and Saving+GVNS gap increases but the average gap remains under 0.49% which again verifies the robustness of our Saving+GVNS algorithm. Over the instances with 100 nodes, the maximum Saving+GVNS gap is 1.68% in the 100-R9 instance with 5 HSPTs. Similar to the instances with 50 nodes, we can observe that the CRT decreases when the number of HSPTs increases. The average CRT over the 10 instances with 5 HSPTs is 29.91 s and it lowers to 17.01 and 15.04 in the instances of 8 and 10 HSPTs, respectively. When the number of HSPTs increases, the achieved best objective function value decreases. However, on average, when the number of HSPTs increases from 5 to 8, the objective function has a decrease of 21.40% and when the number of HSPTs increases from 8 to 10, the average decrease in the objective function is 7.51%. This shows that increasing the number of HSPTs beyond a certain point might not decrease the objective function significantly.

Table 11 gives the results of testing the Saving+GVNS algorithm on instances with 200 and 500 nodes. These instances are again adopted from Salehipour et al. (2011) and a uniformly distributed random number between 1 and 5 is assigned to each node as its weight.

The results of the instances with 200 nodes are given on the left hand side of the Table 11. For each of these instances, we set the number of HSPTs 5, 8, 10. The average CRT over these instances is 289.32 s with a maximum of 660.85 s on 200-R5 instance with 5 HSPTs. The average Saving+GVNS gap for the instances with 200 nodes is 1.60%. The average CRT over instances with 5, 8 and 10 HSPTs are 1.84, 1.82 and 1.35%, respectively. This confirms the robustness of the Saving+GVNS algorithm.

For the instances with 500 nodes, we tested each instance with 10, 15 and 20 HSPTs and gave the results on the right side of Table 11. The average CRT over these instances is 1080.66 s. The maximum CRT is up to 1869.74 s for the 500-R10 instance with 10 HSPTs. The average CRT decreases as the number of HSPTs increases. With 10, 15 and 20 HSPTs, the average CRT is 1724.12, 902.37 and 615.48 s, respectively. The average Saving+GVNS gap is 2.17% and this average gap has a decreasing trend as the number of HSPTs increases. While for the instances with 500 nodes and 10 HSPTs, the average Saving+GVNS gap is 3.36%, this average gap decreases to 1.79% for 15 HSPTs and 1.36% with 20 HSPTs.

**Table 11:** Large instances results for instances with 200 and 500 nodes

$n = 200$						$n= 500$					
Instance	*m*	Best OFV	Avg OFV	CRT	Saving+GVNS GAP (%)	Instance	*m*	Best OFV	Avg OFV	CRT	Saving+GVNS GAP (%)
200-R1	5	61,212	62,828.1	434.21	2.64	500-R1	10	691,005	722,804.1	1783.11	4.60
	8	44,900	45,671.5	181.07	1.72		15	515,484	524,198.9	925.52	1.69
	10	39,955	40,414.3	138.44	1.15		20	434,111	440,058.8	582.57	1.37
200-R2	5	67,182	68,543.7	558.40	2.03	500-R2	10	735,485	761,131.4	1610.59	3.49
	8	51,690	52,392.4	202.81	1.36		15	566,237	579,711.4	852.42	2.38
	10	48,093	48,361.7	210.71	0.56		20	496,613	502,221.8	587.06	1.13
200-R3	5	59,235	60,775.3	425.02	2.60	500-R3	10	735,437	755,097.1	1699.91	2.67
	8	42,175	43,411.8	314.02	2.93		15	570,233	586,216.0	870.08	2.80
	10	37,965	38,523.1	131.43	1.47		20	513,739	518,261.0	651.17	0.88
200-R4	5	62,796	63,792.9	442.88	1.59	500-R4	10	691,517	711,198.6	1674.71	2.85
	8	44,891	45,543.6	169.09	1.45		15	517,098	525,151.1	878.46	1.56
	10	40,204	40,538.4	190.63	0.83		20	449,809	454,880.0	610.82	1.13
200-R5	5	57,839	58,654.5	660.85	1.41	500-R5	10	680,424	696,743.4	1731.13	2.40
	8	40,066	40,965.3	188.99	2.24		15	504,144	516,053.4	883.58	2.36
	10	34,930	35,222.2	160.22	0.84		20	435,699	442,946.9	614.97	1.66
200-R6	5	62,943	64,273.2	586.97	2.11	500-R6	10	705,411	719,660.4	1702.11	2.02
	8	42,660	43,464.2	185.79	1.89		15	545,831	552,813.2	909.86	1.28
	10	36,387	36,966.6	117.06	1.59		20	470,859	476,279.2	580.21	1.15
200-R7	5	60,127	60,856.3	411.55	1.21	500-R7	10	666,005	700,416.9	1725.30	5.17
	8	40,546	41,209.0	210.72	1.64		15	514,699	521,397.1	913.35	1.30
	10	34,949	35,677.8	137.88	2.09		20	448,275	455,944.3	593.79	1.71
200-R8	5	57,533	58,193.4	506.60	1.15	500-R8	10	735,993	755,161.2	1718.36	2.60
	8	40,496	41,343.4	235.48	2.09		15	570,012	578,983.5	893.57	1.57
	10	35,758	36,214.5	148.47	1.28		20	498,810	506,144.4	720.95	1.47
200-R9	5	62,912	64,055.3	555.95	1.82	500-R9	10	648,364	681,186.5	1726.25	5.06
	8	45,856	46,643.9	190.50	1.72		15	498,085	505,992.0	955.05	1.59
	10	41,090	41,523.3	119.01	1.05		20	415,308	423,022.1	609.03	1.86
200-R10	5	57,291	58,371.2	499.54	1.89	500-R10	10	713,622	733,410.5	1869.74	2.77
	8	44,532	45,032.6	214.76	1.12		15	553,981	561,590.1	941.83	1.37
	10	41,127	41,331.3	150.47	0.50		20	487,962	493,889.5	604.24	1.21
Average				289.32	1.60	Average				1080.66	2.17
